# Machine learning-based approach for disease severity classification of carpal tunnel syndrome

**DOI:** 10.1038/s41598-021-97043-7

**Published:** 2021-08-31

**Authors:** Dougho Park, Byung Hee Kim, Sang-Eok Lee, Dong Young Kim, Mansu Kim, Heum Dai Kwon, Mun-Chul Kim, Ae Ryoung Kim, Hyoung Seop Kim, Jang Woo Lee

**Affiliations:** 1 Department of Rehabilitation Medicine, Pohang Stroke and Spine Hospital, Pohang, Republic of Korea; 2 Department of Orthopedic Surgery, Pohang Stroke and Spine Hospital, Pohang, Republic of Korea; 3 Department of Neurosurgery, Pohang Stroke and Spine Hospital, Pohang, Republic of Korea; 4 Department of Rehabilitation Medicine, Kyungpook National University School of Medicine, Kyungpook National University Hospital, Daegu, Republic of Korea; 5 Department of Physical Medicine and Rehabilitation, National Health Insurance Service Ilsan Hospital, Goyang, Republic of Korea

**Keywords:** Neurological disorders, Electrodiagnosis

## Abstract

Identifying the severity of carpal tunnel syndrome (CTS) is essential to providing appropriate therapeutic interventions. We developed and validated machine-learning (ML) models for classifying CTS severity. Here, 1037 CTS hands with 11 variables each were retrospectively analyzed. CTS was confirmed using electrodiagnosis, and its severity was classified into three grades: mild, moderate, and severe. The dataset was randomly split into a training (70%) and test (30%) set. A total of 507 mild, 276 moderate, and 254 severe CTS hands were included. Extreme gradient boosting (XGB) showed the highest external validation accuracy in the multi-class classification at 76.6% (95% confidence interval [CI] 71.2–81.5). XGB also had an optimal model training accuracy of 76.1%. Random forest (RF) and k-nearest neighbors had the second-highest external validation accuracy of 75.6% (95% CI 70.0–80.5). For the RF and XGB models, the numeric rating scale of pain was the most important variable, and body mass index was the second most important. The one-versus-rest classification yielded improved external validation accuracies for each severity grade compared with the multi-class classification (mild, 83.6%; moderate, 78.8%; severe, 90.9%). The CTS severity classification based on the ML model was validated and is readily applicable to aiding clinical evaluations.

## Introduction

The carpal tunnel comprises carpal bones and transverse carpal ligaments. Nine flexor tendons and the median nerve pass through the tunnel^1^. Carpal tunnel syndrome (CTS), the most common entrapment neuropathy, occurs when the median nerve is compressed within the carpal tunnel. CTS presents various clinical manifestations ranging from mild pain to thenar muscle weakness or atrophy depending on the degree and duration of neural compression^2^. Therefore, it is important to properly diagnose CTS severity and determine appropriate treatment options according to the severity grade^3^.

Electrodiagnosis is the gold standard test for diagnosing peripheral nerve diseases and plays an essential role in diagnosing CTS^4,5^. This technique is advantageous for confirming CTS and grading its severity^6–8^. Additionally, differential diagnoses for cervical radiculopathies and other neuropathies can also be conformed with electrodiagnosis. However, owing to electrical stimulation and needle electromyography (EMG) during examinations, electrodiagnosis is invasive and can cause discomfort to the patient^9,10^.

Machine-learning (ML)-based modeling is an emerging analysis tool. It is mainly utilized for implementing predictive models in medical research^11,12^. Furthermore, ML-based modeling can be applied in disease classification, decision-making, and developing new therapeutic interventions^13,14^. However, despite the explosive growth in ML-based medical research, research on CTS is relatively sparse. Although some studies have investigated prediction models for CTS diagnosis^15^, an ML-based model for classifying CTS severity based on comprehensive clinical data has not yet been presented. Therefore, this study evaluated new classification models for determining electrodiagnostic CTS severity using ML algorithms. We also identified the importance of variables to the performance of the ML-based CTS severity classification model.

## Results

### Baseline characteristics

Table 1 shows the summary of all variables and their baseline values. The results of the post-hoc analysis for all continuous variables is shown in Table S1. Among the 1037 hands, 507 (48.9%) were mild, 276 (26.6%) were moderate, and 254 (24.5%) were severe grade.

**Table 1:** Variables and baseline characteristics.

	Electrodiagnostic severity			P value
	Mild	Moderate	Severe	
Hands, n (%)	507 (48.9)	276 (26.6)	254 (24.5)	–
Age in years, mean ± SD	57.3 ± 10.6	59.2 ± 10.8	57.8 ± 11.2	0.069^a^
Male sex, n (%)	199 (39.2)	123 (44.6)	83 (32.7)	0.183^b^
Body-mass index in kg/m^2^, mean ± SD	24.2 ± 3.4	24.7 ± 3.0	25.8 ± 3.7	< 0.001^a^
Right side involvement, n (%)	264 (52.1)	127 (46.0)	135 (53.1)	0.960^b^
Diabetes, n (%)	47 (9.3)	45 (16.3)	54 (21.6)	< 0.001^b^
Duration in months, mean ± SD	4.3 ± 5.0	8.5 ± 8.2	15.9 ± 12.8	< 0.001^a^
Numeric rating scale of pain, mean ± SD	3.3 ± 1.3	4.9 ± 1.5	6.1 ± 1.5	< 0.001^a^
Nocturnal pain, n (%)	102 (20.1)	142 (51.4)	212 (83.5)	< 0.001^b^
Thenar weakness and/or atrophy, n (%)	1 (0.2)	24 (8.7)	169 (66.5)	< 0.001^b^
CSA in mm^2^, mean ± SD	13.2 ± 3.0	15.4 ± 3.2	18.9 ± 5.0	< 0.001^a^
PB of flexor retinaculum in mm, mean ± SD	2.1 ± 0.8	2.6 ± 2.4	3.1 ± 2.3	< 0.001^a^

SD, standard deviation; CSA, cross-sectional area; PB, palmar bowing.

^a^One-way analysis of variance.

^b^Chi-square trend test.

Considering the demographic data, the patients in the moderate grade were oldest (59.2 ± 10.8 years old), and the mild grade were the youngest (57.3 ± 10.6 years old). The difference in age between the groups was not statistically significant (P = 0.069). The proportion of males was relatively low in all severity grades (mild, 39.2%; moderate, 44.6%; severe, 32.7%; P = 0.183). The involvement side was bilaterally distributed; no difference was observed between grades (P = 0.960). The severity of the disease increased with body mass index (BMI), where mild, moderate, and severe grade patients had BMIs of 24.2 ± 3.4, 24.7 ± 3.0, and 25.8 ± 3.7 kg/m^2^, respectively. Further, there were statistically significant differences between all groups (P < 0.001) except among the mild and moderate groups. The occurrence of diabetes was higher in patients with a severe condition; 21.6% individuals in the severe grade had diabetes (P < 0.001).

Duration of symptoms was 4.3 ± 5.0, 8.5 ± 8.2, 15.9 ± 12.8 months for mild, moderate, and severe grade, respectively, and the differences were statistically significant (P < 0.001). Similarly, with higher severity, the numeric rating scale of pain (NRS) was significantly higher (P < 0.001). Additionally, 20.1% patients in the mild grade complained of night pain, followed by 51.4% in the moderate grade and 83.5% in the severe grade; here again, we observed a significant increase with grade severity (P < 0.001). Thenar muscle weakness and/or atrophy was rarely observed in the mild grade, and the rate was low (8.7%) for the moderate grade. However, the proportion of patients complaining of thenar muscle weakness and/or atrophy significantly increased to 66.5% in the severe grade (P < 0.001).

Sonographic findings showed that both cross-sectional area (CSA) of the median nerve and palmar bowing (PB) of the flexor retinaculum increased with disease severity. Statistically significant differences were observed between all the grades in CSA (P < 0.001) and PB (P < 0.001).

### Multi-class classification

We assessed optimal model training performance and validated each ML algorithm utilized for multi-class classification (Table 2). Among the ML algorithms evaluated, the extreme gradient boosting (XGB) had the highest accuracy, with an accuracy of 76.1% during training and 76.6% for test prediction (95% confidence interval [CI] 71.2–81.5). Considering balanced accuracy, XGB had an accuracy of 83.6% and 83.5% for mild and severe grades, and 71.8% for moderate grade. Further, random forest (RF) and k-nearest neighbors (KNN) had the second-highest test prediction accuracy at 75.6% (95% CI 70.0–80.5) with optimal model training accuracies of 76% (RF) and 71.6% (KNN), respectively. Moreover, all the ML models had an accuracy of 70% or higher, and the balanced accuracy of the moderate grade was relatively low compared with the mild and severe grades. The confusion matrix of external validation with XGB, RF, and KNN is presented in Table 3. The best test prediction result of XGB showed the highest sensitivity of 89.8% in the mild grade and the highest specificity of 97.5% in the severe grade. In the severe grade, the test prediction results using XGB also demonstrated the best positive and negative predictive values (90.9% and 90.0%, respectively).

**Table 2:** Results of optimal training model performance and test prediction of each machine learning algorithm for multi-class classification.

Classifier	Training model	Test prediction	Balanced accuracy by class		
	Accuracy, %	Overall accuracy, % (95% CI)	Mild, %	Moderate, %	Severe, %
Neural Network	72.7	73.7 (68.1–78.8)	83.6	66.3	84.3
Support Vector Machines	73.0	74.5 (68.9–79.5)	82.9	68.5	81.4
k-Nearest Neighbors	71.6	75.6 (70.0–80.5)	82.5	64.2	84.4
Classification And Regression Tree	73.6	70.8 (65.0–76.1)	78.1	61.8	81.7
Random Forest	76.0	75.6 (70.0–80.5)	83.6	70.5	81.9
Stochastic Gradient Boosting	74.7	73.4 (67.7–78.5)	80.3	71.6	81.6
eXtreme Gradient Boosting	76.1	76.6 (71.2–81.5)	83.6	71.8	83.5

CI, confidence interval.

**Table 3:** Confusion matrix of three-best performed algorithms in multi-class classification.

Algorithms	Predicted class	Actual class		
		Mild	Moderate	Severe
eXtreme Gradient Boosting	Mild	123	23	8
	Moderate	14	37	14
	Severe	0	5	50
Random Forest	Mild	123	23	8
	Moderate	14	36	16
	Severe	0	6	48
k-Nearest Neighbors	Mild	131	36	6
	Moderate	6	25	15
	Severe	0	4	51

**Figure 1:**
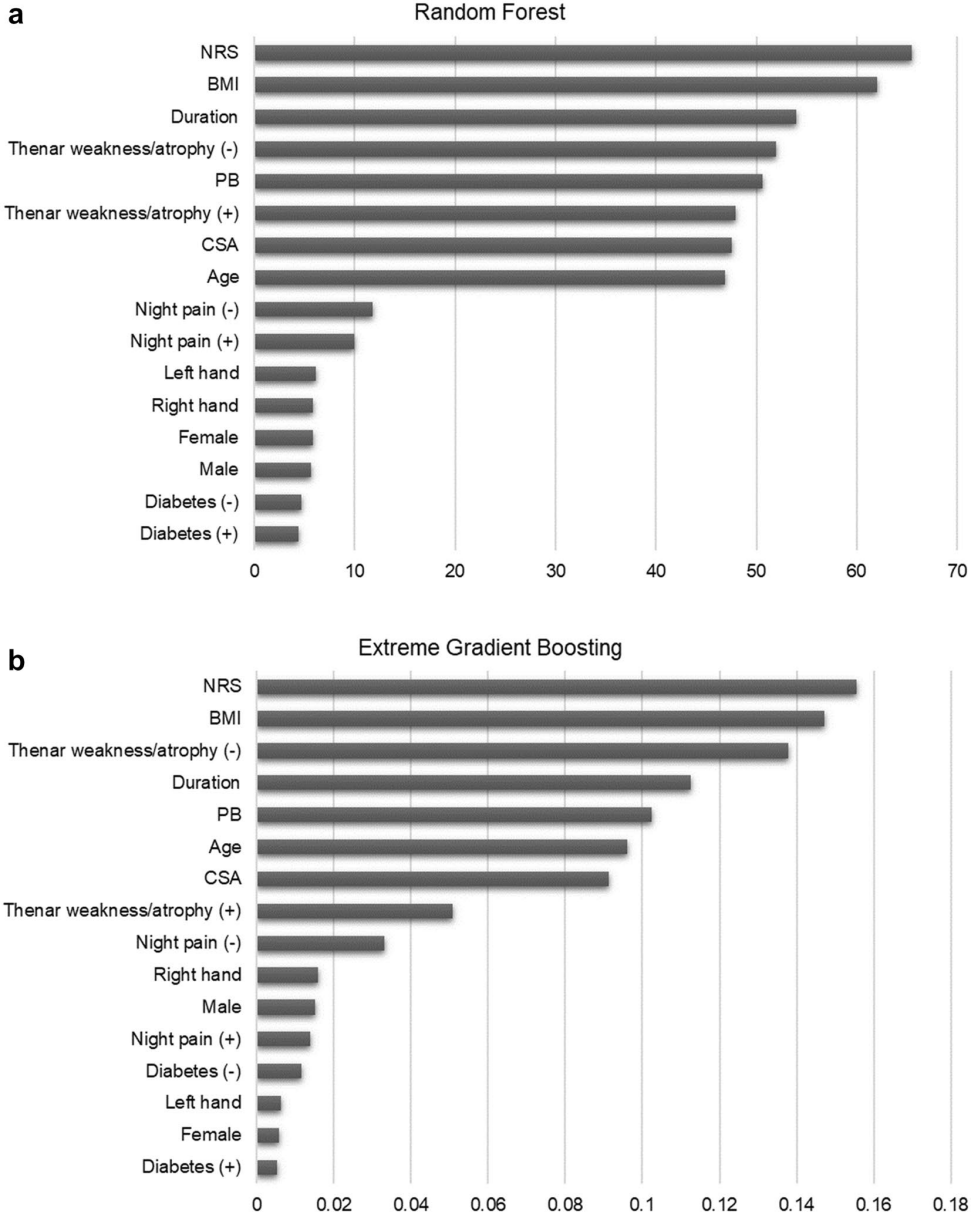
Variable importance. Features that allowed high model performance were determined for RF (**a**) and XGB models (**b**). NRS, BMI, symptom duration, no thenar weakness or atrophy, and PB were among the top five features in both cases. NRS, numeric rating scale of pain; BMI, body-mass index; PB, palmar bowing; CSA, cross-sectional area; RF, random forest; XGB, extreme gradient boosting.

Considering variable importance, the rank of the top five important variables in the RF model were as follows: NRS, BMI, symptom duration, no thenar weakness and atrophy, and PB. In the XGB model, the top five important variables were NRS, BMI, no thenar weakness and atrophy, symptom duration, and PB. The ranking showed that both models selected the same variables as important in almost the same order. Both models selected NRS as the most important variable. Sex, diabetes, involved side, and night pain were selected as variables of low importance (Fig. 1).

### One-versus-rest classification

The best model performances of the one-versus-rest classification using a stacked algorithm for each severity grade are summarized in Table 4, and the relevant confusion matrix for each grade is shown in Table 5. By performing these analyses, we could improve the test prediction accuracy compared with the multi-class classification. The test prediction accuracy was 83.6% (95% CI 78.7–87.8) for mild grade, 78.8% (95% CI 73.5–83.5) for moderate grade, and 90.9% (95% CI 86.8–94.0) for severe grade. The optimal training model for the severe grade showed the highest receiver operating characteristics (ROC) value (0.95). The ROC values of the mild and moderate grades were 0.86 and 0.81, respectively. The mild grade showed high sensitivity (86.1%), but specificity remained relatively low (81.0%). In contrast, in the moderate and severe grades, sensitivity was low, but specificity was relatively high (84.7% and 97.0%, respectively). The severe grade showed the best positive and negative predictive values (89.8% and 91.2%, respectively). The entire results of the one-versus-rest classification are shown in Table S2.

**Table 4:** Best test prediction results of stacked machine-learning algorithms for one-versus-rest classification.

	Mild	Moderate	Severe
ROC	0.86	0.81	0.95
Accuracy, % (95% CI)	83.6 (78.7–87.8)	78.8 (73.5–83.5)	90.9 (86.8–94.0)
Sensitivity, %	86.1	60.0	73.6
Specificity, %	81.0	84.7	97
Positive predictive value, %	81.9	54.9	89.8
Negative predictive value, %	85.4	87.2	91.2

ROC, receiver operating characteristics; CI, confidence interval.

**Table 5:** Confusion matrix of the best test prediction results in each severity grade.

Severity grades	Prediction	Actual class	
		Target	Others
Mild	Target	118	26
	Others	19	111
Moderate	Target	39	32
	Others	26	177
Severe	Target	53	6
	Others	19	196

## Discussion

In this study as with electrodiagnostic techniques, we employed an ML-base modeling approach to investigate the feasibility of identifying CTS severity based on personal, clinical, and sonographic features. All ML models yielded higher than 70% accuracy, and the XGB model performed best. Furthermore, the one-versus-rest classification improved the accuracy compared to the multiclass classification.

CTS has a wide spectrum of symptoms and signs^16^. Because therapeutic options may vary according to its severity, it is important to determine appropriate severity grading of CTS^17,18^. Electrodiagnosis remains the main evaluation tool for CTS severity grading. It is believed that CTS severity grading based on nerve conduction studies provides well-correlated results with the clinical findings^6,19^. However, we developed a modified CTS severity grading system based on Stevens et al.^7^ and utilized it as our basis because we judged that the sensitivity might be low, particularly for the severe grade if the severity was evaluated without EMG. When neural compression progresses, EMG reveals evidence of axonal denervation^20^. Sustained neural compression can lead either to muscle atrophy or weakness^21^. Therefore, needle EMG in the CTS hand acts as an important evaluation tool, allowing surgeons and physicians to decide whether early surgical decompression is required before clinical presentation of thenar muscle atrophy or weakness. However, needle EMG has the disadvantage of causing discomfort to the patient, and owing to its invasive nature, it presents risks of bleeding and infection^9,10^. Our ML-based classification system predicted the electrodiagnosis-based severity grade by utilizing the patient's basic information, clinical information, and non-invasive sonography results. Therefore, our classification model alongside selective electrodiagnostic evaluation, which can help the surgeon or physician to effectively determine CTS severity with minimal discomfort to the patient, is of clinical significance.

Previous studies have applied ML models to CTS assessment. In a recent study by Faeghi et al.^22^, sonographic images at the wrist level were obtained from CTS and control groups. The images underwent segmentation processes, and the accuracy of CTS diagnosis was analyzed based on the ML modeling. They reported that the diagnostic accuracy of radiologists improved when computer-aided diagnosis was applied. Sayin et al.^23^ applied four ML algorithms (i.e., support vector machine, naive Bayes, classification tree, artificial neural network) to 109 CTS patients and 42 control subjects for CTS detection. They demonstrated a CTS detection score of 91.0%. However, these previous studies were limited in their study design because they only suggested the presence or absence of CTS. As mentioned, CTS represents a variety of symptoms and signs according to severity. Therefore, confirmation of severity grade is essential to the determination of treatment options. Meanwhile, Wei et al.^24^ also reported the ML-base CTS assessment. They identified that hand kinematic features were important for CTS diagnosis and severity grading using RF in controls, mild-, and moderate-CTS hands. In their study, although the kinematic features comprised a potential predictor in grading CTS severity, consideration of clinical aspects were lacking. In contrast, we comprehensively assessed the basic personal factors, subjective findings, and objective findings as variables. Consequently, our study design and results are more useful because they mirror the clinical practice of evaluating CTS. We also have the advantage of analyzing the largest number of CTS hands compared with the aforementioned studies.

XGB is superior to other ML models for building prediction models based on regression or classification^25,26^. Here, multi-class classification suggested that the XGB model had the highest accuracy. This finding is consistent with previous studies. XGB, which utilizes the ensemble boosting technique, improves the slow learning speed of gradient boosting and prevents overfitting through regularized training^26,27^. In addition, XGB is the model-of-choice in ML-based research because it can control various hyperparameters, and it is highly flexible^28^. RF is a tree-based ensemble classification model that corrects the overfitting problem by using the bagging method^29,30^. In our analyses, the model accuracy was second only to XGB. These ensemble ML techniques enable classification and prediction of clinical data in a feasible and robust manner^31^.

We identified the variable importance according to our high-performing ML algorithms (i.e., RF and XGB). Both models provided an almost identical order for the top-five variables of importance. Cazares-Manríquez et al.^32^ reported that age, femininity, and high BMI were risk factors for CTS. Our results confirmed that among these personal risk factors, BMI was primarily involved in ordinal severity classification. However, among the variables of high importance, there was no statistically significant difference in BMI between the mild and moderate grades. Moreover, in line with previous studies^33,34^, NRS and symptom duration were also identified as important clinical factors. Furthermore, based on our models, NRS was determined as the most important variable. Therefore, we suggest that the degree of pain subjectively felt by the patient is correlated with electrodiagnostic severity. Additionally, thenar muscle weakness and/or atrophy was obtained as another variable of high importance. We believe that symptoms act as a distinguishing feature between severe and other grades. Sonographic findings, such as CSA and PB, also had high importance. These findings are known to be correlated with CTS severity^35^. Most studies related to sonography and CTS severity focused on CSA^36–38^; however, our data suggested that PB was more important than CSA for classification.

In multi-class classification, the moderate grade had lower balanced accuracy than other grades. This may be because, compared with other grades, moderate CTS has higher clinical diversity among them. The proportion of patients with thenar weakness and/or atrophy was only 8.7% in moderate grade, and this relatively low value may increase error rates. However, compared with the multi-class classification, the one-versus-rest classification provided higher accuracy for the moderate grade. Because NRS and sonographic findings showed high variable importance and represented serial and gradual differences by severity, we might derive better results than those of multi-class classification in one-versus-rest classification for the moderate grade. Moreover, compared with the multi-class classification, we obtained improved accuracy with high specificity for the severe grade as well. In particular, the high specificity of the severe grade is thought to be attributable to relatively important variables, such as thenar muscle weakness and/or atrophy and symptom duration, which were distinguishably different in the severe grade. Therefore, we believe our findings can play a supportive role in the clinic by allowing surgeons to determine CTS severity and decide surgical treatment accordingly.

Another reason for better performance in one-versus-rest classification for the moderate and severe grades is that performing synthetic minority oversampling techniques (SMOTE) to reduce target class imbalance may further improve model performance. SMOTE is widely used as a balancing method to minimize the overfitting frequently encountered by the random up-sampling method^39^. SMOTE also has the advantage of no information loss when extracting a subset of data from the minor class and creating new similar instances by utilizing the KNN algorithm. However, while generating the synthetic data of the minor class, the adjacent instances of the major class are not considered; thus, it cannot be efficient for high-dimensional data^40^. Our target class ratio was mildly imbalanced (approximately 2:1:1 for mild, moderate, and severe grades, respectively). When the up-sampling method was applied to multi-class classification, we found that overfitting occurred. Therefore, a suitable model was not generated. In contrast, in one-versus-rest classification, we created the best performance model with SMOTE for both moderate and severe grades.

We also applied the algorithm stacking method in our binary classifications. It has been found through previous studies that the stacked ML algorithm model can reduce the classification error rate and perform better predictions than the single ML algorithm model^41–43^. We expected these findings to be generalized to the CTS severity classification model when designing our ML processes. In our results, the stacking ML-algorithm models provided better optimal training and prediction results, consistent with previous studies. When combining the performance of each ML-algorithms, we applied the generalized linear model (GLM), RF, and XGB. GLM is the preferred simple blend method for combination and has the advantage of low possibility of overfitting^44^. Meanwhile, RF and XGB are ensembled algorithms that showed the best classification performance in our multi-class classification. Therefore, they were also used as the combination methods for our one-versus-rest classification. Because they are more complex than the simple linear method, they can provide a finely tuned model. However, the more sophisticated the combination method, the more susceptible it is to overfitting^44^. In our stacked algorithms, the combination method for the optimal model was different for each grade. Therefore, it is important to find the best stacking method for the optimized model when combining the predictions of each while reducing overfitting.

Our study has some limitations. This was a retrospective study. Although the dataset was collected at a single center, an inter-clinician bias caused by the relatively long sampling period may have occurred. The dataset used in the study had a relatively small number of disease-related variables, and environmental factors related to overuse were not available. Additionally, we did not have access to long-term follow-up data. These limitations in our data may have affected classification performance.

In conclusion, the ML-based CTS severity classification is readily applicable based on our internal and external validation results. ML-based models performed well when classifying mild and severe grades. In contrast, model accuracies were relatively low when classifying the moderate grade. Among the ML algorithms evaluated, XGB had the best performance, and the variables, particularly NRS, provided high classification accuracy. Therefore, surgeons and physicians can utilize our novel ML-based classification model to make better therapeutic decisions for patients with CTS.

## Methods

### Study design and variables

The dataset was retrospectively collected from a single center between January 2015 and February 2021. Patients diagnosed with CTS by electrodiagnostic evaluation were considered, and we used the following personal variables: age, sex, involved side, BMI, and diabetes incidence. In addition, we evaluated symptoms at the patient’s first visit (e.g., duration of symptoms, NRS, nocturnal pain, and thenar muscle weakness and/or atrophy), which were used as clinical variables. The CSA of the median nerve and the PB of the flexor retinaculum were used as ultrasonographic variables. The CSA of the median nerve was measured using transverse images acquired at the level of the pisiform and scaphoid bones^45^. PB was measured at the trapezium and hook of the hamate level^46^. The exclusion criteria were as follows: (1) other concomitant peripheral nerve lesions; (2) concomitant lower cervical radiculopathy; (3) peripheral vascular disease; (4) arthritis (hand or wrist); (5) previous surgical history (wrist or hand); and (6) missing or inconsistent data.

This study was reviewed and approved by the Institutional Review Board of Pohang Stroke and Spine Hospital (Approval No. PSSH0475-202103-HR-012-01) and performed in compliance with the Declaration of Helsinki and the International Conference on Harmonization–Good Clinical Practice Guideline. Due to the retrospective study design, the Institutional Review Board of Pohang Stroke and Spine Hospital allowed the exemption of informed consent.

### Electrodiagnostic evaluation and severity grading

Antidromic median sensory conduction and orthodromic median motor conduction studies were performed. Transcarpal latency (TCL) was measured for CTS diagnosis and values ≥ 1.7 ms were defined as abnormal. If the TCL was normal, CTS-related symptoms were clear, and if TCL was borderline (1.5 ms ≤ TCL < 1.7 ms), we conducted an additional lumbrical-interossei motor or antidromic ring-finger sensory comparison test based on the advice of a physiatrist. Additionally, EMG had been conducted on the abductor pollicis brevis (APB) muscle. Detailed electrodiagnostic techniques and reference values for diagnosing CTS are presented in Table 6^47^. All electrodiagnostic tests were performed using Sierra® wave (Cadwell, Kennewick, WA, USA). Patients were examined in the supine position. The temperature of the electrodiagnosis room was maintained at 25 °C, and the skin temperature was maintained at 32 °C.

**Table 6:** Electrodiagnostic techniques and reference values for diagnosing carpal tunnel syndrome.

	Stimulation site	Recording site	Reference values
**Sensory nerve action potential**			
Median nerve	4 cm proximal to the recording site	2nd digit	Onset latency: ≤ 3.5 ms and peak to peak amplitude: ≥ 20 µV
Transcarpal latency	7 and 14 cm proximal to the recording site, respectively	2nd digit	Difference of onset latency between two stimulations: < 1.7 ms
Ring finger	Median and ulnar nerves at wrist, respectively, 14 cm proximal to the recording site	4th digit	Difference of onset latency between median and ulnar nerves: < 0.6 ms
**Compound motor nerve action potential**			
Median nerve	8 cm proximal to APB muscle	APB muscle	Onset latency: ≤ 4.0 ms and peak to peak amplitude: ≥ 5 mV
Lumbrical/Interossei	Median and ulnar nerves at wrist, respectively	Midpoint of 3rd metacarpal bone	Difference of onset latency between two stimulations: ≤ 0.4 ms

APB, abductor pollicis brevis muscle.

Based on the electrodiagnostic results, we categorized CTS severity into three grades: mild, moderate, and severe. Our grading system is a modified version of the grading scheme introduced by Stevens^7^. Cases with abnormalities in the sensitivity tests, but normal for median compound motor nerve action potential (CMAP) and needle EMG were considered as mild. Cases with abnormal findings in the median CMAP but normal for needle EMG were considered as moderate. Finally, the severe grade included patients who experienced denervation potentials or polyphasic, long duration, and large amplitude motor unit action potentials in the APB muscle during needle EMG (Table 7).

**Table 7:** Severity grading system according to the electrodiagnostic findings.

Defined grade	Electrodiagnostic findings
Mild	Abnormality in any sensitivity tests^a^
	And/or abnormal median SNAP^b^
	And normal median CMAP
	And normal EMG finding in APB
Moderate	Abnormality in any sensitivity tests^a^
	And/or abnormal median SNAP^b^
	And abnormal median CMAP^b^
	And normal EMG finding in APB
Severe	Abnormality in any sensitivity tests^a^
	And/or abnormal median SNAP^b^
	And/or abnormal median CMAP^b^
	And positive EMG findings in APB^c^

SNAP, sensory nerve action potential; CMAP, compound motor nerve action potential; EMG, electromyography; APB, abductor pollicis brevis muscle.

^a^Transcarpal latency, lumbrical/interossei motor comparison study, or antidromic ring finger sensory comparison study.

^b^Prolonged onset latency or low amplitude.

^c^Denervation potentials or polyphasic, long duration, and large amplitude motor unit action potentials.

### Data analysis

Continuous variables were expressed as mean ± standard deviation, while categorical variables were indicated as frequencies (proportions). To identify the differences in baseline characteristics between each electrodiagnostic severity grade, we conducted a one-way analysis of variance with the Bonferroni post hoc test. We also conducted a chi-square trend test for the categorical variables. All statistical analyses for the baseline characteristics were performed using SPSS 22.0 (IBM Inc., Armonk, NY, USA).

### Model training and validation

**Figure 2:**
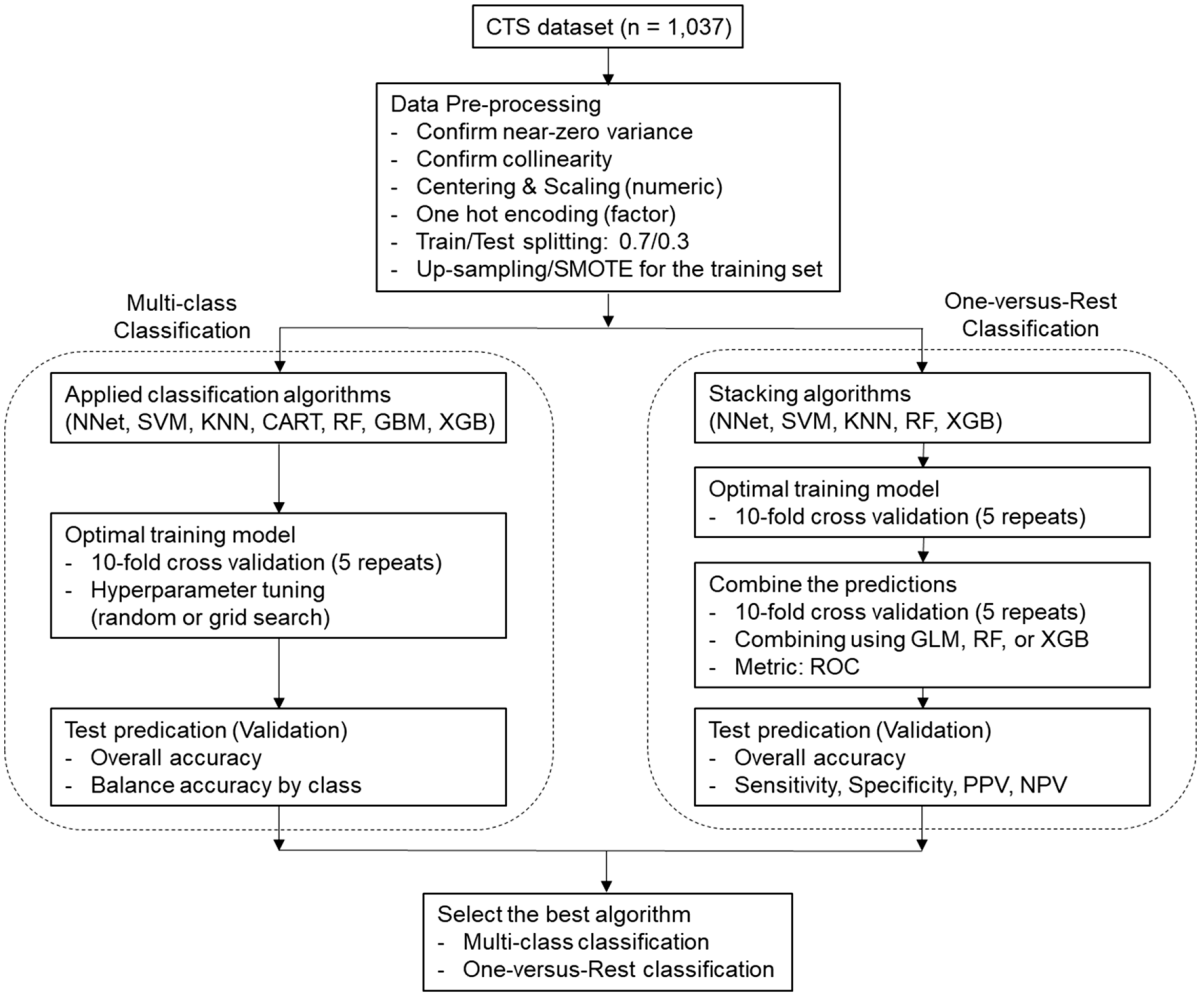
Flowchart of the machine-learning modeling process. CTS, carpal tunnel syndrome; SMOTE, synthetic minority oversampling technique; NNet, neural network; SVM, support vector machines; KNN, k-nearest neighbors; CART, classification and regression tree; RF, random forest; GBM, stochastic gradient boosting; XGB, extreme gradient boosting; GLM, generalized linear model; ROC, receiver operating characteristic; PPV, positive predictive value; NPV, negative predictive value.

All ML processes were performed using R software (version 4.1.0) provided by the R Core Team (R Foundation for Statistical Computing, Vienna, Austria. http://www.R-project.org). The entire dataset and ML processing codes for this study can be found in the online supplementary content. The entire workflow of the ML process in this study is illustrated in Fig. 2.

After exclusion, analysis was conducted using a dataset consisting of 1037 CTS hands. For data pre-processing, we identified variables corresponding to near-zero variance and variables having collinearity. Continuous variables were normalized to zero mean and unit variance values using centering and scaling methods. Categorical variables were encoded using one-hot encoding. The dataset was randomly split into a training and test set at a ratio of 7:3. A total of three levels (i.e., mild, moderate, and severe) were analyzed as target classes. To address an imbalance of target classes in the training set, we utilized the random up-sampling and SMOTE for the multi-class and one-versus-rest classification, respectively.

For the multi-class classification, utilizing the caret package^48^, a total of seven ML algorithms were adopted as follows: neural network, support vector machine, KNN, classification and regression tree, RF, stochastic gradient boosting, and XGB^49–53^. For the training set, we conducted a tenfold cross validation with five repeats to create an optimal training model and utilized either a random or grid search for hyperparameter tuning (Table S3). Additionally, considering the RF model as a representative of the bagging ensemble algorithm and the XGB model as a representative of the boosting ensemble algorithm, we identified the variables of importance. For external validation, we conducted predictions on the test dataset based on the optimal training model created. To evaluate the performance of multi-class classification ML models, we used the overall and balanced accuracies of each class.

We also conducted one-versus-rest classification for each severity grade. We developed a model by stacking five ML algorithms and executed a combined classification utilizing the caretEnsemble package^44^. Five algorithms with different operation mechanisms were selected among those used for multi-class classification. Classification and regression tree and stochastic gradient boosting algorithms were excluded because they share similar operation mechanisms with RF and XGB, respectively. Consequently, the ML classifiers used in the stacked ensemble included the neural network, support vector machine, KNN, RF, and XGB. We conducted a tenfold cross-validation with five repeats to train the stacked ML model. Subsequently, we combined the predictions of each classifier using the GLM, RF, and XGB. Then, we selected the best model among the results from the three combination methods. For external validation, we conducted predictions on the test dataset based on the optimal combined model. We measured the ROC, overall accuracy, sensitivity, specificity, positive predictive value, and negative predictive value to validate the one-versus-rest classification.

## Supplementary Information

Supplementary Information 1.

Supplementary Information 2.

## Data Availability

All data and R codes for this study are included in this published article (and its Supplementary Information files).
