# Supplementary material for: Machine learning-based approach for disease severity classification of carpal tunnel syndrome
Source: Sci Rep. 2021 Aug 31;11:17464. doi: 10.1038/s41598-021-97043-7 (PMC8408248; doi:10.1038/s41598-021-97043-7)
Supplement: Supplementary file 1 — Supplementary Information 1. [file 41598_2021_97043_MOESM1_ESM.pdf]

Machine Learning-Based Approach for Disease Severity Classification of Carpal Tunnel Syndrome

Dougho Park[1], Byung Hee Kim[1], Sang-Eok Lee[1], Dong Young Kim[2], Mansu Kim[3], Heum Dai Kwon[3], Mun-Chul Kim[3], Ae Ryoung Kim[4], Hyoung Seop Kim[5], Jang Woo Lee[5, *]

[1]Department of Rehabilitation Medicine, Pohang Stroke and Spine Hospital, Pohang, Republic of Korea

[2]Department of Orthopedic Surgery, Pohang Stroke and Spine Hospital, Pohang, Republic of Korea

[3]Department of Neurosurgery, Pohang Stroke and Spine Hospital, Pohang, Republic of Korea

[4]Department of Rehabilitation Medicine, Kyungpook National University School of Medicine, Kyungpook National University Hospital, Daegu, Republic of Korea

[5]Department of Physical Medicine and Rehabilitation, National Health Insurance Service Ilsan Hospital, Goyang, Republic of Korea

*medipia@gmail.com

Table S1. Results of post-hoc analysis for baseline characteristics.

	Electrodiagnosis severity			P value[a]	P value[b]	P value[c]
	Mild	Moderate	Severe			
Age, years (mean ± SD)	57.3 ± 10.6	59.2 ± 10.8	57.8 ± 11.2	0.065	> 0.999	0.349
BMI, kg/m2 (mean ± SD)	24.2 ± 3.4	24.7 ± 3.0	25.8 ± 3.7	0.136	< 0.001	0.001
Duration, months (mean ± SD)	4.3 ± 5.0	8.5 ± 8.2	15.9 ± 12.8	< 0.001	< 0.001	< 0.001
NRS (mean ± SD)	3.3 ± 1.3	4.9 ± 1.5	6.1 ± 1.5	< 0.001	< 0.001	< 0.001
CSA, mm2 (mean ± SD)	13.2 ± 3.0	15.4 ± 3.2	18.9 ± 5.0	< 0.001	< 0.001	< 0.001
PB, mm (mean ± SD)	2.1 ± 0.8	2.6 ± 2.4	3.1 ± 2.3	0.001	< 0.001	< 0.001

Abbreviations: SD, standard deviation; BMI, body mass index; NRS, numeric rating scale of pain; CSA, cross-sectional area; PB, palmar bowing.

[a]mild *vs.* moderate

[b]mild *vs.* severe

[c]moderate *vs.*severe

Table S2. Entire results of one-versus-rest classification.

Combination	Metric	Mild	Moderate	Moderate (SMOTE)	Severe	Severe (SMOTE)
RF	ROC	0.86	0.61	0.81	0.91	0.96
	Accuracy (95% CI)	82.1 (77.1–86.5)	78.1 (72.7–82.9)	78.8[a] (73.5–83.5)	90.9 (86.8–94.0)	89.4 (85.2–92.8)
	Sensitivity	86.1	27.7	60.0	69.4	66.7
	Specificity	78.1	93.8	84.7	98.5	97.5
	PPV	79.7	58.1	54.9	94.3	90.6
	NPV	84.9	80.7	87.2	90.1	89.1
GLM	ROC	0.86	0.62	0.79	0.89	0.95
	Accuracy (95% CI)	83.6[a] (78.7–87.8)	78.8 (73.5–83.5)	78.1 (72.7–82.9)	90.5 (86.4–93.7)	90.2 (86.0–93.4)
	Sensitivity	86.1	23.1	53.9	69.4	69.4
	Specificity	81.0	96.2	85.7	98.0	97.5
	PPV	81.9	65.2	53.9	92.6	90.9
	NPV	85.4	80.0	85.7	90.0	90.0
XGB	ROC	0.85	0.62	0.79	0.9	0.95
	Accuracy (95% CI)	82.9 (77.9–87.1)	78.1 (72.7–82.9)	78.5 (73.1–83.2)	89.8 (85.6–93.1)	90.9[a] (86.8–94.0)
	Sensitivity	88.3	16.9	58.5	66.7	73.6
	Specificity	77.4	97.1	84.7	98.0	97.0
	PPV	79.6	64.7	54.3	92.3	89.8
	NPV	86.9	79.0	86.8	89.2	91.2

Abbreviations: SMOTE, synthetic minority oversampling technique; RF, random forest; GLM, generalized linear model; XGB, extreme gradient boosting; ROC, receiver operating characteristics; CI, Confidence Interval; PPV, positive predictive value; NPV, negative predictive value.

[a]the best test prediction result in each severity grade

Table S3. Optimal parameters of each classification algorithms.

	Algorithms	Optimal parameters
Multi-class	Neural Network	size = 5, decay = 0.1
	Support Vector Machines	sigma = 0.05584847, cost = 4
	k-Nearest Neighbors	k (neighbors) = 27
	Classification and Regression Tree	complexity parameter = 0.02035623
	Random Forest	n.trees = 500, mtry = 9
	Stochastic Gradient Boosting	n.trees = 300, interaction.depth = 8, shrinkage = 0.1, n.minobsinnode = 10
	eXtreme Gradient Boosting	nrounds = 500, max_depth = 7, eta = 0.01, gamma = 0, colsample_bytree = 0.6, min_child_weight = 1, subsample = 0.8
One-versus-rest	Generalized linear model (combination)	Not applicable
	Random Forest (combination)	n.trees = 500, mtry = 2
	eXtreme Gradient Boosting (combination)	nrounds = 150, max_depth = 3, eta = 0.3, gamma = 0, colsample_bytree = 0.6, min_child_weight = 1 and subsample = 1

Classification of carpal tunnel syndrome severity grade

(Revised Version)

Dougho Park

2021 07 08

R Markdown

```
library(caret)
library(caretEnsemble)
library(doParallel)
library(data.table)
library(dplyr)
library(e1071)
library(gbm)
library(kernlab)
library(randomForest)
library(tidyverse)
library(xgboost)
library(smotefamily)
```

■ *Road the dataset and variable definition*

```
CTS <- read_csv("CTS.csv")
CTS$Severity<-as.factor(CTS$Severity)
CTS$Mild<-as.factor(CTS$Mild)
CTS$Mod<-as.factor(CTS$Mod)
CTS$Sev<-as.factor(CTS$Sev)
CTS$Sex <-as.factor(CTS$Sex)
CTS$Side <-as.factor(CTS$Side)
CTS$Diabetes <-as.factor(CTS$Diabetes)
CTS$NP <- as.factor(CTS$NP)
CTS$Weakness <- as.factor(CTS$Weakness)
```

■ *Data Pre-processing*

```
predata<-CTS

nearZeroVar(predata)

## integer(0)
```

```
findCorrelation(cor(predata[,5:10]), cutoff = .75)

## integer(0)

st_model<-preProcess(predata[,5:10], method=c("center","scale"))
data<-predict(st_model, predata)

data=as.data.frame(data)
ohe_feats = c('Sex','Side','Diabetes','NP','Weakness')
dummies = dummyVars(~ Sex+Side+Diabetes+NP+Weakness, data = data)
df_ohe <- as.data.frame(predict(dummies, newdata = data))
df_combined <- cbind(data[,-c(which(colnames(data) %in% ohe_feats))],df_ohe)
dat = as.data.table(df_combined)
```

■ *Data splitting*

```
seed<-0923
set.seed(seed)
ind<-sample(2,nrow(dat),replace = T,prob = c(0.7,0.3))
traindata<-dat[ind==1,]
testdata<-dat[ind==2,]

trainmc<-traindata
testmc<-testdata

trainmc$Mild=NULL
trainmc$Mod=NULL
trainmc$Sev=NULL
testmc$Mild=NULL
testmc$Mod=NULL
testmc$Sev=NULL
```

■ *Mutli-class classification*

```
ctrl <- trainControl(method="repeatedcv", number=10, repeats=5)
metric <- "Accuracy"
```

- *Neural Network_ Training model*

```
set.seed(seed)
nn_fit <- train(Severity ~., data = trainmc,
                method = "nnet",
```

```
                trControl=ctrl, metric=metric,
                tuneLength = 5, verbose=FALSE)
```

```
nn_fit
```

```
## Neural Network
##
## 763 samples
##  16 predictor
##   3 classes: 'mild', 'moderate', 'severe'
##
## No pre-processing
## Resampling: Cross-Validated (10 fold, repeated 5 times)
## Summary of sample sizes: 687, 686, 687, 687, 687, 686, ...
## Resampling results across tuning parameters:
##
##   size  decay  Accuracy   Kappa
##   1     0e+00  0.7192462  0.5426980
##   1     1e-04  0.7186960  0.5427086
##   1     1e-03  0.7202885  0.5448820
##   1     1e-02  0.7124245  0.5308880
##   1     1e-01  0.7071750  0.5204891
##   3     0e+00  0.7040346  0.5272508
##   3     1e-04  0.7124794  0.5367610
##   3     1e-03  0.7126508  0.5364463
##   3     1e-02  0.7174252  0.5473359
##   3     1e-01  0.7211091  0.5518733
##   5     0e+00  0.6935359  0.5100833
##   5     1e-04  0.6964812  0.5141826
##   5     1e-03  0.6914643  0.5044617
##   5     1e-02  0.6983163  0.5148666
##   5     1e-01  0.7268406  0.5598330
##   7     0e+00  0.6714475  0.4758963
##   7     1e-04  0.6843556  0.4942923
##   7     1e-03  0.6903985  0.5057582
##   7     1e-02  0.6918059  0.5074657
##   7     1e-01  0.7098720  0.5347687
##   9     0e+00  0.6754763  0.4834766
##   9     1e-04  0.6733811  0.4813848
##   9     1e-03  0.6843792  0.4958223
##   9     1e-02  0.6791199  0.4877426
##   9     1e-01  0.6982691  0.5161380
##
## Accuracy was used to select the optimal model using the largest value.
## The final values used for the model were size = 5 and decay = 0.1.
```

```
plot(nn_fit)
```

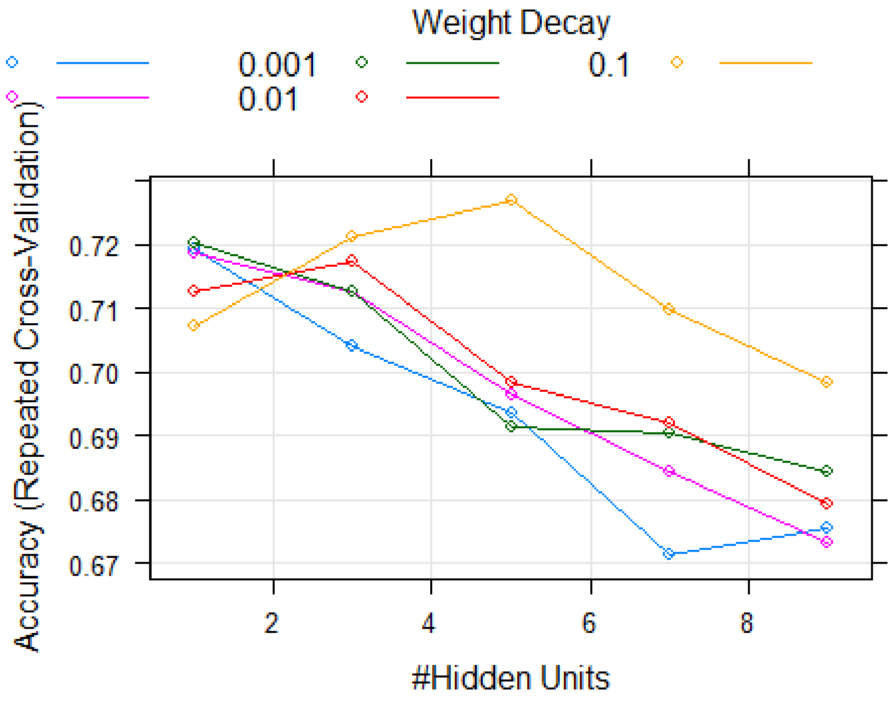

- ***Neural Network_ Test prediction***

```
test_pred_nn <- predict(nn_fit, newdata = testmc)
confusionMatrix (test_pred_nn, testmc$Severity)
```

```
## Confusion Matrix and Statistics
##
##             Reference
## Prediction mild moderate severe
##   mild       116       22      2
##   moderate    21       33     17
##   severe       0       10     53
##
## Overall Statistics
##
##                Accuracy : 0.7372
##                  95% CI : (0.6809, 0.7883)
##     No Information Rate : 0.5
##     P-Value [Acc > NIR] : 9.512e-16
##
##                   Kappa : 0.578
##
##  Mcnemar's Test P-Value : 0.2795
##
## Statistics by Class:
##
```

```
##                         Class: mild Class: moderate Class: severe
## Sensitivity                  0.8467          0.5077        0.7361
## Specificity                  0.8248          0.8182        0.9505
## Pos Pred Value               0.8286          0.4648        0.8413
## Neg Pred Value               0.8433          0.8424        0.9100
## Prevalence                   0.5000          0.2372        0.2628
## Detection Rate               0.4234          0.1204        0.1934
## Detection Prevalence         0.5109          0.2591        0.2299
## Balanced Accuracy            0.8358          0.6629        0.8433
```

- ***Support Vector Machines_Training model***

```
set.seed(seed)
svm_fit <- train(Severity ~., data = trainmc,
                 method = "svmRadial",
                 trControl=ctrl, metric=metric,
                 tuneLength = 10)
svm_fit

## Support Vector Machines with Radial Basis Function Kernel
##
## 763 samples
##  16 predictor
##   3 classes: 'mild', 'moderate', 'severe'
##
## No pre-processing
## Resampling: Cross-Validated (10 fold, repeated 5 times)
## Summary of sample sizes: 687, 686, 687, 687, 687, 686, ...
## Resampling results across tuning parameters:
##
##   C       Accuracy   Kappa
##     0.25  0.7032754  0.5113243
##     0.50  0.7056336  0.5144520
##     1.00  0.7145536  0.5304672
##     2.00  0.7245197  0.5488446
##     4.00  0.7297489  0.5592372
##     8.00  0.7291921  0.5602135
##    16.00  0.7181873  0.5433293
##    32.00  0.7058325  0.5244693
##    64.00  0.6909007  0.5018361
##   128.00  0.6817311  0.4885673
##
## Tuning parameter 'sigma' was held constant at a value of 0.05584847
## Accuracy was used to select the optimal model using the largest value.
## The final values used for the model were sigma = 0.05584847 and C = 4.

plot(svm_fit)
```

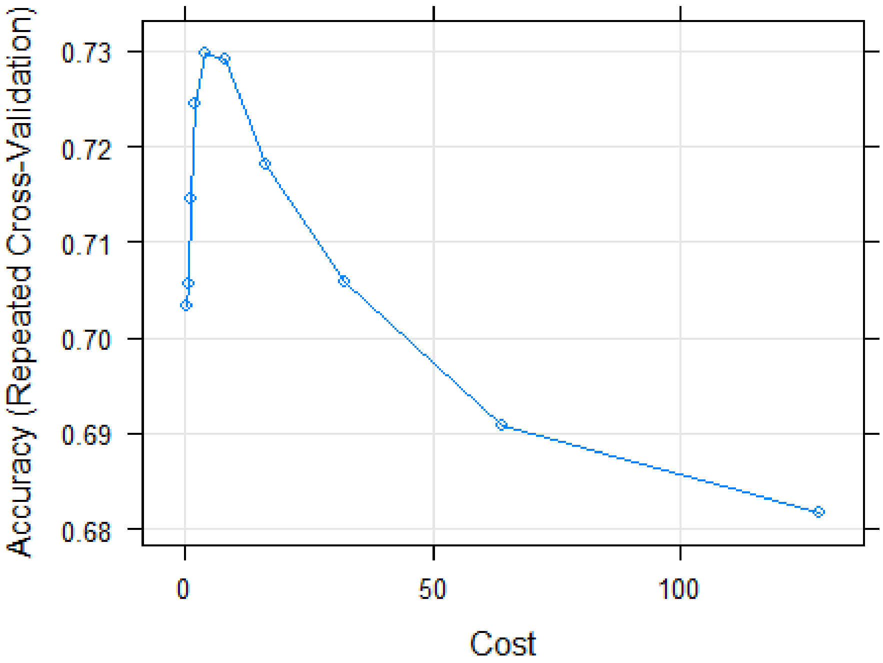

- ***Support Vector Machines_Test prediction***

```
test_pred_svm <- predict(svm_fit, newdata = testmc)
confusionMatrix (test_pred_svm, testmc$Severity)
```

```
## Confusion Matrix and Statistics
##
##             Reference
## Prediction mild moderate severe
##   mild      123       26      7
##   moderate   14       34     18
##   severe      0        5     47
##
## Overall Statistics
##
##                Accuracy : 0.7445
##                  95% CI : (0.6886, 0.7951)
##     No Information Rate : 0.5
##     P-Value [Acc > NIR] : < 2.2e-16
##
##                   Kappa : 0.58
##
##  Mcnemar's Test P-Value : 0.0004509
##
## Statistics by Class:
##
```

```
##                      Class: mild Class: moderate Class: severe
## Sensitivity               0.8978          0.5231        0.6528
## Specificity               0.7591          0.8469        0.9752
## Pos Pred Value            0.7885          0.5152        0.9038
## Neg Pred Value            0.8814          0.8510        0.8874
## Prevalence                0.5000          0.2372        0.2628
## Detection Rate            0.4489          0.1241        0.1715
## Detection Prevalence      0.5693          0.2409        0.1898
## Balanced Accuracy         0.8285          0.6850        0.8140
```

- ***k-Nearest Neighbors_Training model***

```
set.seed(seed)
knn_fit <- train(Severity~., data = trainmc,
                 method = "knn",
                 trControl=ctrl, metric=metric,
                 tuneLength=50)
knn_fit
```

```
## k-Nearest Neighbors
##
## 763 samples
##  16 predictor
##   3 classes: 'mild', 'moderate', 'severe'
##
## No pre-processing
## Resampling: Cross-Validated (10 fold, repeated 5 times)
## Summary of sample sizes: 687, 686, 687, 687, 687, 686, ...
## Resampling results across tuning parameters:
##
##   k    Accuracy   Kappa
##     5  0.7032860  0.5188253
##     7  0.6988193  0.5081441
##     9  0.7050837  0.5150279
##    11  0.7132074  0.5274266
##    13  0.7069258  0.5155884
##    15  0.7103026  0.5200821
##    17  0.7132007  0.5236817
##    19  0.7110989  0.5203979
##    21  0.7139800  0.5249987
##    23  0.7153231  0.5267397
##    25  0.7152958  0.5264198
##    27  0.7155452  0.5256795
##    29  0.7103264  0.5164279
##    31  0.7116353  0.5187617
##    33  0.7100633  0.5159219
##    35  0.7056032  0.5077003
##    37  0.7063926  0.5099647
```

```
##    39  0.7061192  0.5091228
##    41  0.7006032  0.4999821
##    43  0.7013892  0.5012280
##    45  0.7019189  0.5021157
##    47  0.7011226  0.5002403
##    49  0.7013926  0.5000543
##    51  0.7040173  0.5040082
##    53  0.7045299  0.5049916
##    55  0.7037439  0.5032246
##    57  0.7042668  0.5042748
##    59  0.7024417  0.5010058
##    61  0.7011362  0.4983902
##    63  0.7006133  0.4973771
##    65  0.7008560  0.4974452
##    67  0.7045334  0.5034198
##    69  0.7029715  0.5001433
##    71  0.7016625  0.4976245
##    73  0.7019257  0.4981075
##    75  0.7006235  0.4954211
##    77  0.7001074  0.4941615
##    79  0.6987883  0.4916572
##    81  0.6987950  0.4911132
##    83  0.6995811  0.4921722
##    85  0.6977527  0.4886245
##    87  0.6977562  0.4880049
##    89  0.6969530  0.4860902
##    91  0.6959004  0.4845393
##    93  0.6966831  0.4855975
##    95  0.6972094  0.4860148
##    97  0.6977391  0.4867358
##    99  0.6943351  0.4805696
##   101  0.6914438  0.4755591
##   103  0.6927664  0.4776843
## 
## Accuracy was used to select the optimal model using the largest value.
## The final value used for the model was k = 27.
```

```
plot(knn_fit)
```

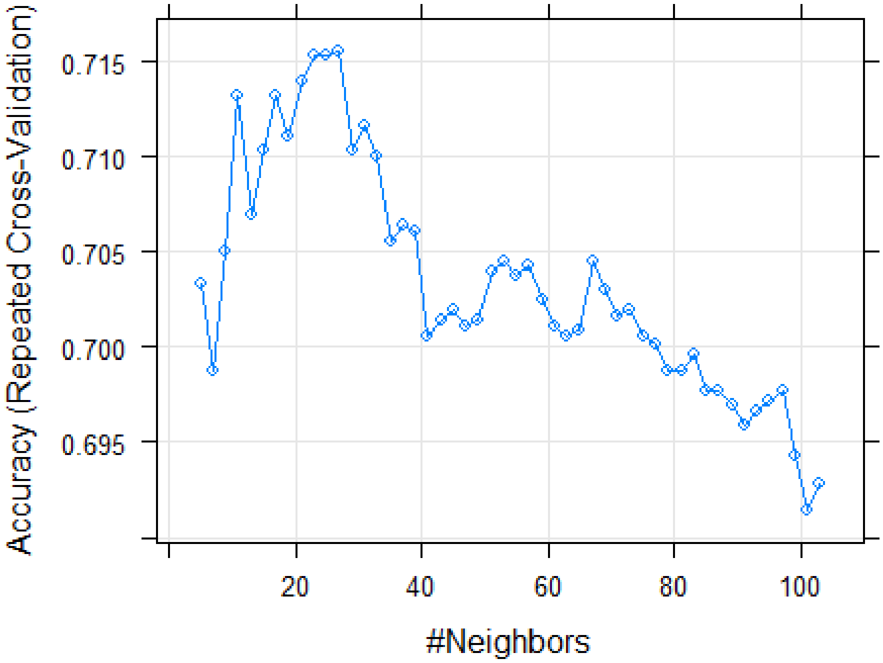

- ***k-Nearest Neighbors_Test prediction***

```
test_pred_knn <- predict(knn_fit, newdata = testmc)
confusionMatrix(test_pred_knn, testmc$Severity)
```

```
## Confusion Matrix and Statistics
##
##             Reference
## Prediction mild moderate severe
##   mild      131       36      6
##   moderate    6       25     15
##   severe      0        4     51
##
## Overall Statistics
##
##                Accuracy : 0.7555
##                  95% CI : (0.7002, 0.8052)
##     No Information Rate : 0.5
##     P-Value [Acc > NIR] : < 2.2e-16
##
##                   Kappa : 0.5868
##
##  Mcnemar's Test P-Value : 2.187e-07
##
## Statistics by Class:
##
```

```
##                      Class: mild Class: moderate Class: severe
## Sensitivity               0.9562         0.38462        0.7083
## Specificity               0.6934         0.89952        0.9802
## Pos Pred Value            0.7572         0.54348        0.9273
## Neg Pred Value            0.9406         0.82456        0.9041
## Prevalence                0.5000         0.23723        0.2628
## Detection Rate            0.4781         0.09124        0.1861
## Detection Prevalence      0.6314         0.16788        0.2007
## Balanced Accuracy         0.8248         0.64207        0.8443
```

- ***Classification And Regression Tree_Training model***

```
set.seed(seed)
rp_fit <- train(Severity ~., data = trainmc,
                method = "rpart",
                trControl=ctrl, metric=metric,
                tuneLength = 10)
rp_fit
```

```
## CART
##
## 763 samples
##  16 predictor
##   3 classes: 'mild', 'moderate', 'severe'
##
## No pre-processing
## Resampling: Cross-Validated (10 fold, repeated 5 times)
## Summary of sample sizes: 687, 686, 687, 687, 687, 686, ...
## Resampling results across tuning parameters:
##
##   cp           Accuracy   Kappa
##   0.003816794  0.7084778  0.5265534
##   0.004240882  0.7095305  0.5280973
##   0.005089059  0.7077190  0.5238921
##   0.006785411  0.7108769  0.5276057
##   0.007633588  0.7244690  0.5499378
##   0.010178117  0.7344416  0.5646792
##   0.020356234  0.7360342  0.5659359
##   0.034351145  0.6983272  0.5075992
##   0.114503817  0.6521713  0.4068928
##   0.307888041  0.5675494  0.1921960
##
## Accuracy was used to select the optimal model using the largest value.
## The final value used for the model was cp = 0.02035623.
```

```
plot(rp_fit)
```

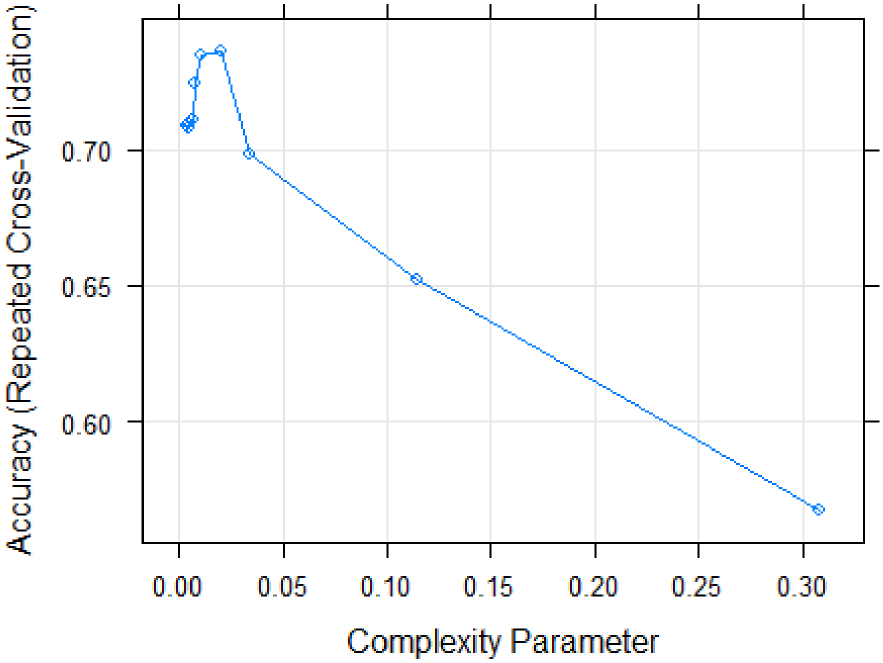

- ***Classification And Regression Tree_Test prediction***

```
test_pred_rp <- predict(rp_fit, newdata = testmc)
confusionMatrix (test_pred_rp, testmc$Severity)
```

```
## Confusion Matrix and Statistics
##
##             Reference
## Prediction mild moderate severe
##   mild      122       36      9
##   moderate   15       25     16
##   severe      0        4     47
##
## Overall Statistics
##
##                Accuracy : 0.708
##                  95% CI : (0.6503, 0.7612)
##     No Information Rate : 0.5
##     P-Value [Acc > NIR] : 2.140e-12
##
##                   Kappa : 0.5116
##
##  Mcnemar's Test P-Value : 1.662e-05
##
## Statistics by Class:
##
```

```
##                      Class: mild Class: moderate Class: severe
## Sensitivity               0.8905         0.38462        0.6528
## Specificity               0.6715         0.85167        0.9802
## Pos Pred Value            0.7305         0.44643        0.9216
## Neg Pred Value            0.8598         0.81651        0.8879
## Prevalence                0.5000         0.23723        0.2628
## Detection Rate            0.4453         0.09124        0.1715
## Detection Prevalence      0.6095         0.20438        0.1861
## Balanced Accuracy         0.7810         0.61815        0.8165
```

- ***Up-Sampling of the training-set***

```
set.seed(seed)
up.train<-upSample(x=trainmc[,-1], y=trainmc$Severity)
table(up.train$Class)
```

```
##
##     mild moderate   severe
##      370      370      370
```

- ***Random Forest_Training model***

```
set.seed(seed)
rf_fit <- train(Severity~., data = trainmc,
                method = "rf", trControl=ctrl, metric=metric,
                tuneLength=15)
rf_fit
```

```
## Random Forest
##
## 763 samples
##  16 predictor
##   3 classes: 'mild', 'moderate', 'severe'
##
## No pre-processing
## Resampling: Cross-Validated (10 fold, repeated 5 times)
## Summary of sample sizes: 687, 686, 687, 687, 687, 686, ...
## Resampling results across tuning parameters:
##
##   mtry  Accuracy   Kappa
##    2    0.7353057  0.5611223
##    3    0.7462767  0.5841515
##    4    0.7507367  0.5931768
##    5    0.7556821  0.6019076
##    6    0.7588196  0.6075904
##    7    0.7549027  0.6016184
```

```
##    8    0.7546397  0.6013336
##    9    0.7598823  0.6102560
##   10    0.7554019  0.6032340
##   11    0.7582830  0.6081369
##   12    0.7543527  0.6018264
##   13    0.7543358  0.6021548
##   14    0.7548620  0.6033910
##   15    0.7543561  0.6024252
##   16    0.7556583  0.6040638
##
## Accuracy was used to select the optimal model using the largest value.
## The final value used for the model was mtry = 9.
```

```
plot(rf_fit)
```

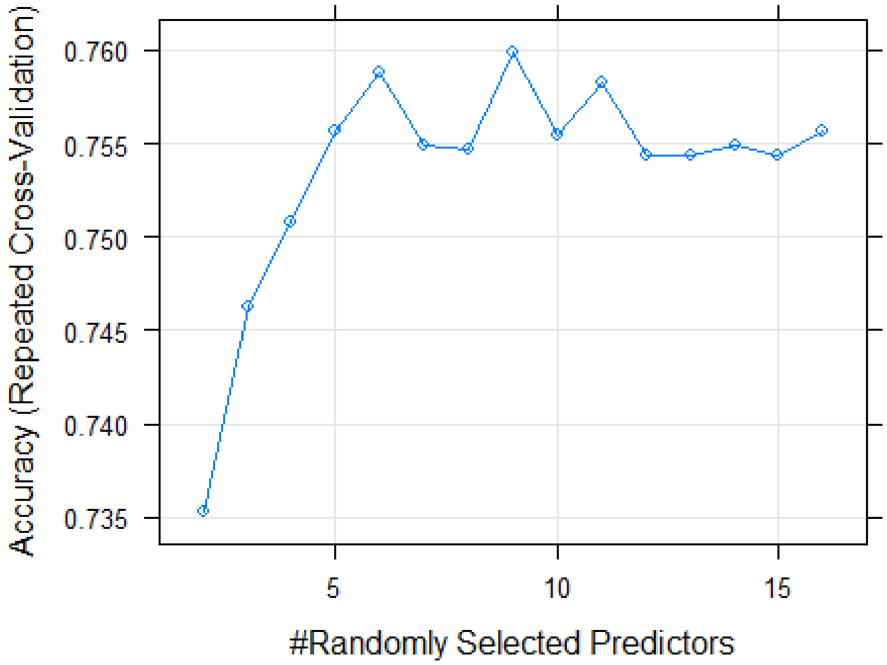

- ***Random Forest_Test prediction***

```
test_pred_rf <- predict(rf_fit, newdata = testmc)
confusionMatrix(test_pred_rf, testmc$Severity)
```

```
## Confusion Matrix and Statistics
##
##             Reference
## Prediction mild moderate severe
##   mild      123       23      8
##   moderate   14       36     16
```

```
##   severe      0        6     48
##
## Overall Statistics
##
##                Accuracy : 0.7555
##                  95% CI : (0.7002, 0.8052)
##     No Information Rate : 0.5
##     P-Value [Acc > NIR] : < 2.2e-16
##
##                   Kappa : 0.5992
##
##  Mcnemar's Test P-Value : 0.002058
##
## Statistics by Class:
##
##                      Class: mild Class: moderate Class: severe
## Sensitivity               0.8978          0.5538        0.6667
## Specificity               0.7737          0.8565        0.9703
## Pos Pred Value            0.7987          0.5455        0.8889
## Neg Pred Value            0.8833          0.8606        0.8909
## Prevalence                0.5000          0.2372        0.2628
## Detection Rate            0.4489          0.1314        0.1752
## Detection Prevalence      0.5620          0.2409        0.1971
## Balanced Accuracy         0.8358          0.7052        0.8185
```

- ***Random Forest_Variable importance***

```
imp<-varImp(rf_fit, scale = FALSE)
imp
```

```
## rf variable importance
##
##             Overall
## NRS          65.434
## BMI          62.056
## Duration     54.016
## Weakness.0   51.947
## PB           50.600
## Weakness.1   47.896
## CSA          47.535
## Age          46.918
## NP.0         11.801
## NP.1         10.006
## Side.1        6.087
## Side.0        5.890
## Sex.1         5.854
## Sex.0         5.700
```

```
## Diabetes.0    4.728
## Diabetes.1    4.439
```

```
plot(imp)
```

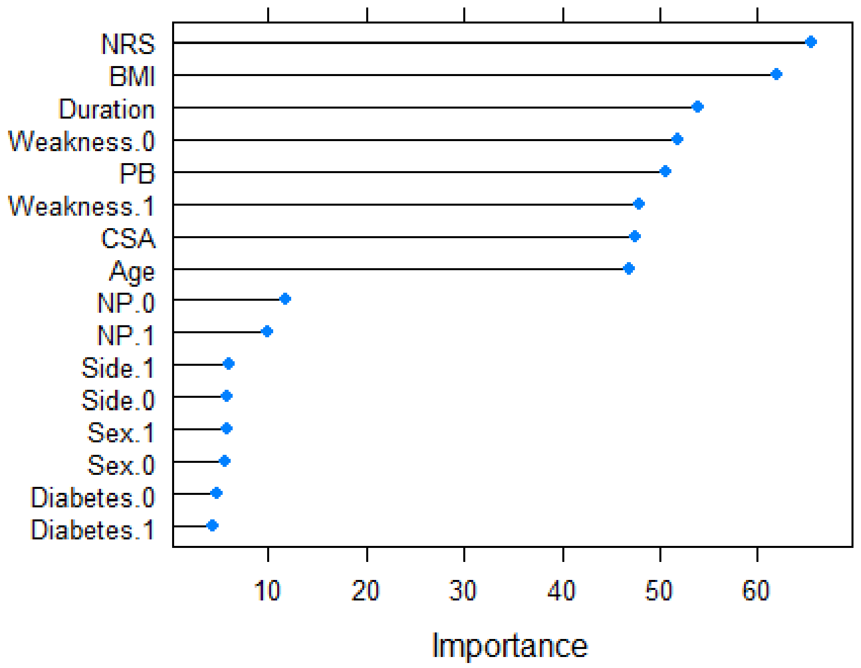

- ***Random Forest_Training model (Up-sampling)***

```
set.seed(seed)
rf_fit_up <- train(Class~., data = up.train,
                method = "rf", trControl=ctrl, metric=metric,
                tuneLength=15)
rf_fit_up
```

```
## Random Forest
##
## 1110 samples
##   16 predictor
##    3 classes: 'mild', 'moderate', 'severe'
##
## No pre-processing
## Resampling: Cross-Validated (10 fold, repeated 5 times)
## Summary of sample sizes: 999, 999, 999, 999, 999, 999, ...
## Resampling results across tuning parameters:
##
##   mtry  Accuracy   Kappa
##    2    0.8410811  0.7616216
```

```
##     3     0.8753153  0.8129730
##     4     0.8834234  0.8251351
##     5     0.8873874  0.8310811
##     6     0.8848649  0.8272973
##     7     0.8868468  0.8302703
##     8     0.8855856  0.8283784
##     9     0.8875676  0.8313514
##    10     0.8879279  0.8318919
##    11     0.8873874  0.8310811
##    12     0.8855856  0.8283784
##    13     0.8879279  0.8318919
##    14     0.8850450  0.8275676
##    15     0.8846847  0.8270270
##    16     0.8881081  0.8321622
##
## Accuracy was used to select the optimal model using the largest value.
## The final value used for the model was mtry = 16.

plot(rf_fit_up)
```

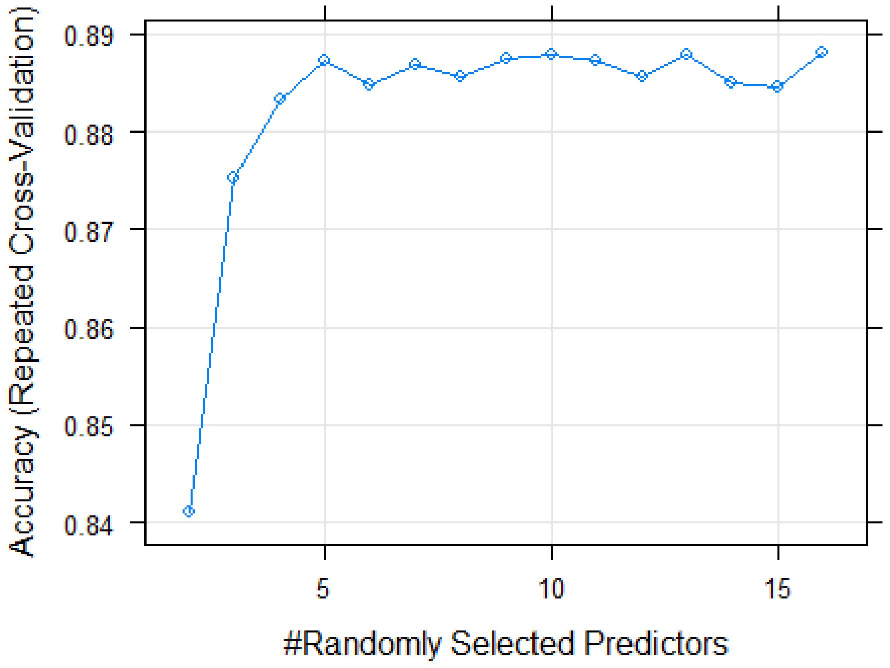

- ***Random Forest_Test prediction (Up-sampling)***

```
test_pred_rf_up <- predict(rf_fit_up, newdata = testmc)
confusionMatrix(test_pred_rf_up, testmc$Severity)
```

```
## Confusion Matrix and Statistics
##
##           Reference
## Prediction mild moderate severe
##   mild      118       18      7
##   moderate   17       39     16
##   severe      2        8     49
##
## Overall Statistics
##
##                Accuracy : 0.7518
##                  95% CI : (0.6963, 0.8018)
##     No Information Rate : 0.5
##     P-Value [Acc > NIR] : <2e-16
##
##                   Kappa : 0.5998
##
##  Mcnemar's Test P-Value : 0.1403
##
## Statistics by Class:
##
##                      Class: mild Class: moderate Class: severe
## Sensitivity               0.8613          0.6000        0.6806
## Specificity               0.8175          0.8421        0.9505
## Pos Pred Value            0.8252          0.5417        0.8305
## Neg Pred Value            0.8550          0.8713        0.8930
## Prevalence                0.5000          0.2372        0.2628
## Detection Rate            0.4307          0.1423        0.1788
## Detection Prevalence      0.5219          0.2628        0.2153
## Balanced Accuracy         0.8394          0.7211        0.8155
```

- ***Stochastic Gradient Boosting_Training model***

```
set.seed(seed)
gbm_fit <- train(Severity~., data = trainmc,
                 method = "gbm",
                 metric=metric, trControl=ctrl,
                 tuneLength=10, verbose=FALSE)
gbm_fit
```

```
## Stochastic Gradient Boosting
##
## 763 samples
##  16 predictor
##   3 classes: 'mild', 'moderate', 'severe'
##
## No pre-processing
## Resampling: Cross-Validated (10 fold, repeated 5 times)
```

```
## Summary of sample sizes: 687, 686, 687, 687, 687, 686, ...
## Resampling results across tuning parameters:
##
##   interaction.depth  n.trees  Accuracy   Kappa
##    1                  50      0.7383957  0.5759624
##    1                 100      0.7376232  0.5770606
##    1                 150      0.7431359  0.5865889
##    1                 200      0.7360512  0.5755661
##    1                 250      0.7349952  0.5746015
##    1                 300      0.7368407  0.5777401
##    1                 350      0.7357811  0.5758300
##    1                 400      0.7339562  0.5730601
##    1                 450      0.7326438  0.5712424
##    1                 500      0.7318442  0.5702140
##    2                  50      0.7417999  0.5825504
##    2                 100      0.7407472  0.5833654
##    2                 150      0.7399441  0.5826607
##    2                 200      0.7354673  0.5753963
##    2                 250      0.7304807  0.5676138
##    2                 300      0.7299614  0.5676042
##    2                 350      0.7312635  0.5694641
##    2                 400      0.7299544  0.5679157
##    2                 450      0.7281191  0.5659371
##    2                 500      0.7254907  0.5617174
##    3                  50      0.7422888  0.5841226
##    3                 100      0.7394009  0.5814307
##    3                 150      0.7307235  0.5684960
##    3                 200      0.7297048  0.5664362
##    3                 250      0.7265570  0.5619401
##    3                 300      0.7291615  0.5660338
##    3                 350      0.7281054  0.5644421
##    3                 400      0.7320323  0.5708451
##    3                 450      0.7296739  0.5670439
##    3                 500      0.7304771  0.5683906
##    4                  50      0.7414857  0.5836429
##    4                 100      0.7328562  0.5714939
##    4                 150      0.7330852  0.5720033
##    4                 200      0.7343941  0.5739504
##    4                 250      0.7336012  0.5730291
##    4                 300      0.7325519  0.5712872
##    4                 350      0.7346639  0.5752222
##    4                 400      0.7357334  0.5772880
##    4                 450      0.7349170  0.5754591
##    4                 500      0.7322923  0.5718469
##    5                  50      0.7417728  0.5847899
##    5                 100      0.7381192  0.5797692
##    5                 150      0.7325962  0.5717401
##    5                 200      0.7349578  0.5761805
##    5                 250      0.7333753  0.5735432
```

```
##    5                    300      0.7333447  0.5733277
##    5                    350      0.7333413  0.5733916
##    5                    400      0.7320255  0.5710274
##    5                    450      0.7354432  0.5766772
##    5                    500      0.7338811  0.5742082
##    6                     50      0.7415096  0.5852886
##    6                    100      0.7391411  0.5814730
##    6                    150      0.7351768  0.5758508
##    6                    200      0.7393971  0.5826540
##    6                    250      0.7401731  0.5839989
##    6                    300      0.7427944  0.5884678
##    6                    350      0.7409695  0.5859291
##    6                    400      0.7406994  0.5848438
##    6                    450      0.7417383  0.5865379
##    6                    500      0.7386078  0.5813874
##    7                     50      0.7422788  0.5865374
##    7                    100      0.7428150  0.5875260
##    7                    150      0.7435941  0.5890561
##    7                    200      0.7425145  0.5874364
##    7                    250      0.7433209  0.5886477
##    7                    300      0.7401630  0.5838107
##    7                    350      0.7427673  0.5881582
##    7                    400      0.7435737  0.5892825
##    7                    450      0.7443394  0.5901356
##    7                    500      0.7427911  0.5883047
##    8                     50      0.7443839  0.5887851
##    8                    100      0.7441308  0.5896602
##    8                    150      0.7464719  0.5935745
##    8                    200      0.7464786  0.5935890
##    8                    250      0.7462189  0.5933297
##    8                    300      0.7467178  0.5939715
##    8                    350      0.7443597  0.5903885
##    8                    400      0.7451357  0.5920395
##    8                    450      0.7438197  0.5895142
##    8                    500      0.7438230  0.5897471
##    9                     50      0.7418065  0.5849690
##    9                    100      0.7367794  0.5776580
##    9                    150      0.7375517  0.5790226
##    9                    200      0.7433309  0.5881928
##    9                    250      0.7407060  0.5843647
##    9                    300      0.7430678  0.5880504
##    9                    350      0.7438368  0.5888383
##    9                    400      0.7430508  0.5878842
##    9                    450      0.7443496  0.5895080
##    9                    500      0.7448726  0.5907972
##   10                     50      0.7443395  0.5889565
##   10                    100      0.7403851  0.5838696
##   10                    150      0.7409354  0.5847833
##   10                    200      0.7420322  0.5864801
```

```
##   10                 250       0.7443665  0.5902925
##   10                 300       0.7425176  0.5867232
##   10                 350       0.7451560  0.5913607
##   10                 400       0.7435840  0.5887917
##   10                 450       0.7438540  0.5888142
##   10                 500       0.7427910  0.5875150
##
## Tuning parameter 'shrinkage' was held constant at a value of 0.1
##
## Tuning parameter 'n.minobsinnode' was held constant at a value of 10
## Accuracy was used to select the optimal model using the largest value.
## The final values used for the model were n.trees = 300, interaction.depth
= 8, shrinkage = 0.1 and n.minobsinnode = 10.
```

```
plot(gbm_fit)
```

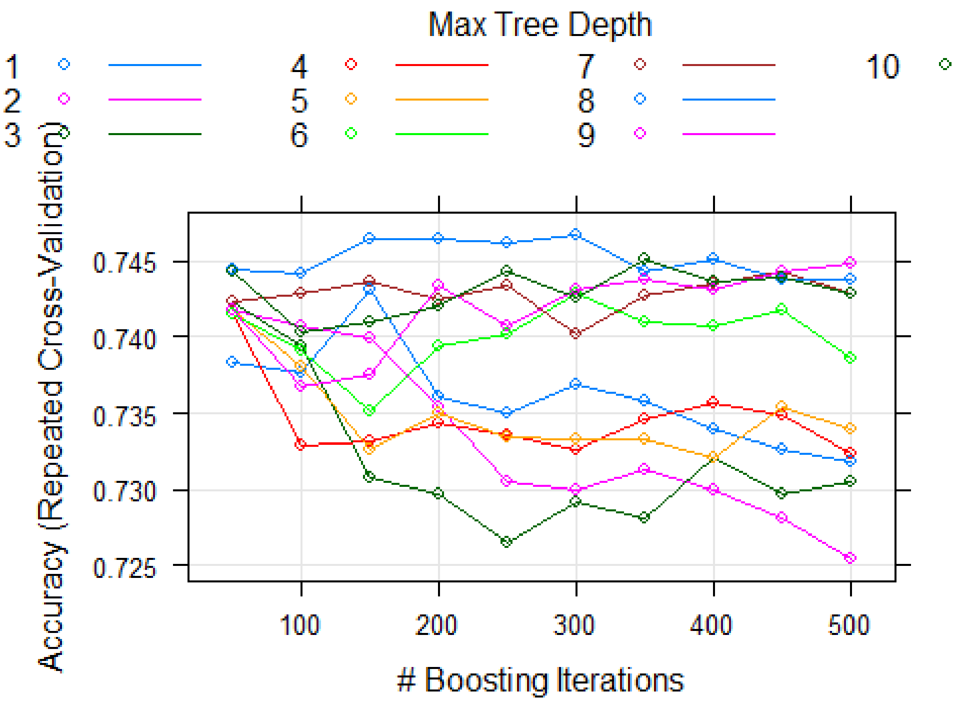

- ***Stochastic Gradient Boosting_Test prediction***

```
test_pred_gbm <- predict(gbm_fit, newdata = testmc)
confusionMatrix(test_pred_gbm, testmc$Severity)
```

```
## Confusion Matrix and Statistics
##
##           Reference
## Prediction mild moderate severe
##   mild      114       20     11
```

```
##   moderate   20       38     12
##   severe      3        7     49
##
## Overall Statistics
##
##                Accuracy : 0.7336
##                  95% CI : (0.6771, 0.785)
##     No Information Rate : 0.5
##     P-Value [Acc > NIR] : 2.659e-15
##
##                   Kappa : 0.569
##
##  Mcnemar's Test P-Value : 0.1172
##
## Statistics by Class:
##
##                      Class: mild Class: moderate Class: severe
## Sensitivity               0.8321          0.5846        0.6806
## Specificity               0.7737          0.8469        0.9505
## Pos Pred Value            0.7862          0.5429        0.8305
## Neg Pred Value            0.8217          0.8676        0.8930
## Prevalence                0.5000          0.2372        0.2628
## Detection Rate            0.4161          0.1387        0.1788
## Detection Prevalence      0.5292          0.2555        0.2153
## Balanced Accuracy         0.8029          0.7158        0.8155
```

- ***eXtreme Gradient Boosting_Training model***

```
tunedxgbgrid <- expand.grid(nrounds=500,
                       max_depth=7,
                       eta=0.01,
                       gamma=0,
                       colsample_bytree=0.6,
                       min_child_weight=1,
                       subsample=0.8)
set.seed(seed)
xgb_fit <- train(Severity~., data=trainmc,
                 method = "xgbTree",
                 metric=metric, trControl=ctrl,
                 tuneGrid=tunedxgbgrid)
xgb_fit
```

```
## eXtreme Gradient Boosting
##
## 763 samples
##  16 predictor
##   3 classes: 'mild', 'moderate', 'severe'
##
```

```
## No pre-processing
## Resampling: Cross-Validated (10 fold, repeated 5 times)
## Summary of sample sizes: 687, 686, 687, 687, 687, 686, ...
## Resampling results:
##
##   Accuracy   Kappa
##   0.7611713  0.6118657
##
## Tuning parameter 'nrounds' was held constant at a value of 500
## Tuning
##  held constant at a value of 1
## Tuning parameter 'subsample' was held
##  constant at a value of 0.8
```

- ***eXtreme Gradient Boosting_Test prediction***

```
test_pred_xgb <- predict(xgb_fit, newdata = testmc)
confusionMatrix(test_pred_xgb, testmc$Severity)

## Confusion Matrix and Statistics
##
##           Reference
## Prediction mild moderate severe
##   mild      123       23      8
##   moderate   14       37     14
##   severe      0        5     50
##
## Overall Statistics
##
##                Accuracy : 0.7664
##                  95% CI : (0.7118, 0.8152)
##     No Information Rate : 0.5
##     P-Value [Acc > NIR] : < 2e-16
##
##                   Kappa : 0.6171
##
##  Mcnemar's Test P-Value : 0.00235
##
## Statistics by Class:
##
##                      Class: mild Class: moderate Class: severe
## Sensitivity               0.8978          0.5692        0.6944
## Specificity               0.7737          0.8660        0.9752
## Pos Pred Value            0.7987          0.5692        0.9091
## Neg Pred Value            0.8833          0.8660        0.8995
## Prevalence                0.5000          0.2372        0.2628
## Detection Rate            0.4489          0.1350        0.1825
```

```
## Detection Prevalence       0.5620          0.2372        0.2007
## Balanced Accuracy          0.8358          0.7176        0.8348
```

- ***eXtreme Gradient Boosting _Variable importance***

```
imp<-varImp(xgb_fit, scale = FALSE)
imp

## xgbTree variable importance
##
##              Overall
## NRS         0.155551
## BMI         0.147273
## Weakness.0 0.137879
## Duration   0.112406
## PB          0.102498
## Age         0.095989
## CSA         0.091232
## Weakness.1 0.050775
## NP.0        0.032985
## Side.0      0.015811
## Sex.0       0.015225
## NP.1        0.013765
## Diabetes.0 0.011545
## Side.1      0.006182
## Sex.1       0.005711
## Diabetes.1 0.005171

plot(imp)
```

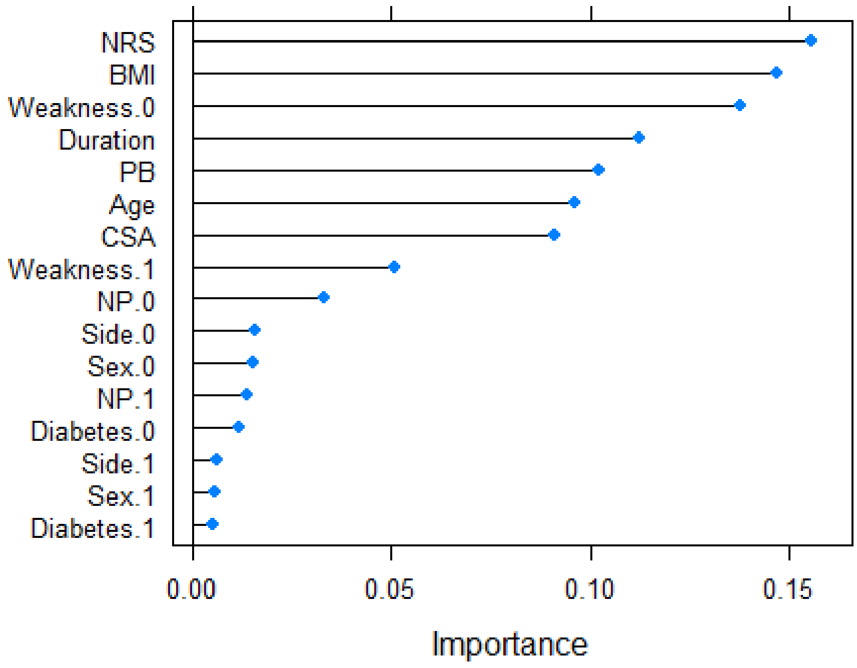

- ***eXtreme Gradient Boosting_Training model (Up-sampling)***

```
tunedxgbgrid_up <- expand.grid(nrounds=500,
                               max_depth=8,
                               eta=0.01,
                               gamma=0,
                               colsample_bytree=0.8,
                               min_child_weight=1,
                               subsample=0.8)
set.seed(seed)
xgb_fit_up <- train(Class~., data=up.train,
                    method = "xgbTree",
                    metric=metric, trControl=ctrl,
                    tuneGrid=tunedxgbgrid_up)
xgb_fit_up

## eXtreme Gradient Boosting
##
## 1110 samples
##   16 predictor
##    3 classes: 'mild', 'moderate', 'severe'
##
## No pre-processing
## Resampling: Cross-Validated (10 fold, repeated 5 times)
## Summary of sample sizes: 999, 999, 999, 999, 999, 999, ...
## Resampling results:
```

```
##
##   Accuracy  Kappa
##   0.883964  0.8259459
##
## Tuning parameter 'nrounds' was held constant at a value of 500
## Tuning
##  held constant at a value of 1
## Tuning parameter 'subsample' was held
##  constant at a value of 0.8
```

- ***eXtreme Gradient Boosting_Test prediction (Up-sampling)***

```
test_pred_xgb_up <- predict(xgb_fit_up, newdata = testmc)
confusionMatrix(test_pred_xgb_up, testmc$Severity)
```

```
## Confusion Matrix and Statistics
##
##             Reference
## Prediction mild moderate severe
##   mild      116       19      5
##   moderate   21       39     15
##   severe      0        7     52
##
## Overall Statistics
##
##                Accuracy : 0.7555
##                  95% CI : (0.7002, 0.8052)
##     No Information Rate : 0.5
##     P-Value [Acc > NIR] : < 2e-16
##
##                   Kappa : 0.6075
##
##  Mcnemar's Test P-Value : 0.04582
##
## Statistics by Class:
##
##                      Class: mild Class: moderate Class: severe
## Sensitivity               0.8467          0.6000        0.7222
## Specificity               0.8248          0.8278        0.9653
## Pos Pred Value            0.8286          0.5200        0.8814
## Neg Pred Value            0.8433          0.8693        0.9070
## Prevalence                0.5000          0.2372        0.2628
## Detection Rate            0.4234          0.1423        0.1898
## Detection Prevalence      0.5109          0.2737        0.2153
## Balanced Accuracy         0.8358          0.7139        0.8438
```

■ *Data extraction for mild-versus-rest classification*

```
trainmild<-traindata
testmild<-testdata

trainmild$Severity=NULL
trainmild$Mod=NULL
trainmild$Sev=NULL
testmild$Severity=NULL
testmild$Mod=NULL
testmild$Sev=NULL

table(trainmild$Mild)
```

```
##
##   mild others
##    370    393
```

```
registerDoParallel(5)
getDoParWorkers()
```

```
## [1] 5
```

- *Stacking algorithms*

```
ovrctrl <- trainControl(method="repeatedcv", number=10, repeats=5,
                        savePredictions='final',
                        classProbs=TRUE,
                        summaryFunction = twoClassSummary,
                        allowParallel=TRUE,
                        index = createFolds(trainmild$Mild, 10))
metric <- "ROC"
algorithmList <- c('rf', 'knn', 'svmRadial', 'nnet', 'xgbTree')

set.seed(seed)
model_mild <- caretList(Mild~., data=trainmild,
                        metric=metric,
                        trControl=ovrctrl,
                        methodList= algorithmList,
                        tuneList = NULL,
                        continue_on_fail = FALSE)
```

```
## # weights:  55
## initial  value 519.277064
## iter  10 value 307.117742
## iter  20 value 283.600593
```

```
## iter  30 value 271.634050
## iter  40 value 266.343071
## iter  50 value 262.414504
## iter  60 value 261.939463
## iter  70 value 261.785497
## final  value 261.778490
## converged
```

```
result_mild <- resamples(model_mild)
summary(result_mild)
```

```
##
## Call:
## summary.resamples(object = result_mild)
##
## Models: rf, knn, svmRadial, nnet, xgbTree
## Number of resamples: 10
##
## ROC
##                Min.   1st Qu.    Median      Mean   3rd Qu.      Max. NA's
## rf        0.8682199 0.8733840 0.8756926 0.8787788 0.8816685 0.8972277    0
## knn       0.8236310 0.8436349 0.8527044 0.8487675 0.8564455 0.8631890    0
## svmRadial 0.8453793 0.8518434 0.8590333 0.8583766 0.8653208 0.8684362    0
## nnet      0.8490757 0.8538185 0.8581505 0.8616138 0.8629477 0.8933211    0
## xgbTree   0.8431482 0.8598683 0.8631409 0.8660625 0.8710568 0.8951629    0
##
## Sens
##                Min.   1st Qu.    Median      Mean   3rd Qu.      Max. NA's
## rf        0.7807808 0.8295796 0.8528529 0.8447447 0.8633634 0.8918919    0
## knn       0.7837838 0.8325826 0.8378378 0.8504505 0.8813814 0.9099099    0
## svmRadial 0.7177177 0.7364865 0.7552553 0.7783784 0.8273273 0.8798799    0
## nnet      0.6996997 0.7545045 0.7777778 0.7762763 0.8093093 0.8228228    0
## xgbTree   0.6846847 0.7695195 0.8033033 0.7888889 0.8160661 0.8528529    0
##
## Spec
##                Min.   1st Qu.    Median      Mean   3rd Qu.      Max. NA's
## rf        0.6892655 0.7258066 0.7542373 0.7441454 0.7669972 0.7740113    0
## knn       0.6045198 0.6546610 0.7043901 0.6833765 0.7166058 0.7308782    0
## svmRadial 0.6742210 0.7330508 0.7736952 0.7576951 0.7929311 0.8022599    0
## nnet      0.7146893 0.7650326 0.7768362 0.7789176 0.8035303 0.8192090    0
## xgbTree   0.6977401 0.7372881 0.7861190 0.7707287 0.8060891 0.8276836    0
```

```
stctrl <- trainControl(method="repeatedcv", number=10, repeats=5,
                       savePredictions=TRUE,
                       classProbs=TRUE,
                       summaryFunction = twoClassSummary,
                       allowParallel=TRUE,
                       index = createFolds(trainmild$Mild, 10))
```

- ***Combine Predictions with Random Forest***

```
set.seed(seed)
stack.rf_mild <- caretStack(model_mild, method="rf", metric="ROC", trControl=
stctrl)
print(stack.rf_mild)
```

```
## A rf ensemble of 5 base models: rf, knn, svmRadial, nnet, xgbTree
##
## Ensemble results:
## Random Forest
##
## 6867 samples
##    5 predictor
##    2 classes: 'mild', 'others'
##
## No pre-processing
## Resampling: Cross-Validated (10 fold, repeated 5 times)
## Summary of sample sizes: 76, 76, 77, 76, 76, 76, ...
## Resampling results across tuning parameters:
##
##   mtry  ROC        Sens       Spec
##   2     0.8594460  0.6799600  0.8416900
##   3     0.8572017  0.6713238  0.8423757
##   5     0.8531713  0.6664865  0.8384068
##
## ROC was used to select the optimal model using the largest value.
## The final value used for the model was mtry = 2.
```

```
test_pred_mild <- predict(stack.rf_mild, newdata = testmild)
confusionMatrix(test_pred_mild, testmild$Mild)
```

```
## Confusion Matrix and Statistics
##
##           Reference
## Prediction mild others
##     mild    118     30
##     others   19    107
##
##                Accuracy : 0.8212
##                  95% CI : (0.7706, 0.8647)
##     No Information Rate : 0.5
##     P-Value [Acc > NIR] : <2e-16
##
##                   Kappa : 0.6423
##
##  Mcnemar's Test P-Value : 0.1531
##
```

```
##              Sensitivity : 0.8613
##              Specificity : 0.7810
##           Pos Pred Value : 0.7973
##           Neg Pred Value : 0.8492
##               Prevalence : 0.5000
##           Detection Rate : 0.4307
##     Detection Prevalence : 0.5401
##        Balanced Accuracy : 0.8212
##
##         'Positive' Class : mild
##
```

- ***Combine Predictions with Generalized Linear Model***

```
set.seed(seed)
stack.glm_mild <- caretStack(model_mild, method="glm", metric="ROC", trContro
l=stctrl)
print(stack.glm_mild)
```

```
## A glm ensemble of 5 base models: rf, knn, svmRadial, nnet, xgbTree
##
## Ensemble results:
## Generalized Linear Model
##
## 6867 samples
##    5 predictor
##    2 classes: 'mild', 'others'
##
## No pre-processing
## Resampling: Cross-Validated (10 fold, repeated 5 times)
## Summary of sample sizes: 76, 76, 77, 76, 76, 76, ...
## Resampling results:
##
##   ROC        Sens      Spec
##   0.8609651  0.678425  0.8418186
```

```
test_pred_mild_glm <- predict(stack.glm_mild, newdata = testmild)
confusionMatrix(test_pred_mild_glm, testmild$Mild)
```

```
## Confusion Matrix and Statistics
##
##           Reference
## Prediction mild others
##     mild    118     26
##     others   19    111
##
##                Accuracy : 0.8358
##                  95% CI : (0.7865, 0.8776)
```

```
##     No Information Rate : 0.5
##     P-Value [Acc > NIR] : <2e-16
##
##                   Kappa : 0.6715
##
##  Mcnemar's Test P-Value : 0.3711
##
##             Sensitivity : 0.8613
##             Specificity : 0.8102
##          Pos Pred Value : 0.8194
##          Neg Pred Value : 0.8538
##              Prevalence : 0.5000
##          Detection Rate : 0.4307
##    Detection Prevalence : 0.5255
##       Balanced Accuracy : 0.8358
##
##        'Positive' Class : mild
##
```

- ***Combine Predictions with eXtreme Gradient Boosting***

```
set.seed(seed)
stack.xgb_mild <- caretStack(model_mild, method="xgbTree", metric="ROC", trCo
ntrol=stctrl)
print(stack.xgb_mild)
```

```
## A xgbTree ensemble of 5 base models: rf, knn, svmRadial, nnet, xgbTree
##
## Ensemble results:
## eXtreme Gradient Boosting
##
## 6867 samples
##    5 predictor
##    2 classes: 'mild', 'others'
##
## No pre-processing
## Resampling: Cross-Validated (10 fold, repeated 5 times)
## Summary of sample sizes: 76, 76, 77, 76, 76, 76, ...
## Resampling results across tuning parameters:
## Tuned ROC: 0.3  1          0.8              1.00        50      0.8475596
## ## Tuning parameter 'gamma' was held constant at a value of 0
## Tuning
##  parameter 'min_child_weight' was held constant at a value of 1
## ROC was used to select the optimal model using the largest value.
## The final values used for the model were nrounds = 50, max_depth = 1, eta
##  = 0.3, gamma = 0, colsample_bytree = 0.8, min_child_weight = 1 and subsam
ple = 1.
```

```
test_pred_mild_xgb <- predict(stack.xgb_mild, newdata = testmild)
confusionMatrix(test_pred_mild_xgb, testmild$Mild)

## Confusion Matrix and Statistics
##
##             Reference
## Prediction mild others
##     mild     121     31
##     others    16    106
##
##                Accuracy : 0.8285
##                  95% CI : (0.7785, 0.8712)
##     No Information Rate : 0.5
##     P-Value [Acc > NIR] : < 2e-16
##
##                   Kappa : 0.6569
##
##  Mcnemar's Test P-Value : 0.04114
##
##             Sensitivity : 0.8832
##             Specificity : 0.7737
##          Pos Pred Value : 0.7961
##          Neg Pred Value : 0.8689
##              Prevalence : 0.5000
##          Detection Rate : 0.4416
##    Detection Prevalence : 0.5547
##       Balanced Accuracy : 0.8285
##
##        'Positive' Class : mild
##
```

■ *Data extraction for moderate-versus-rest classification*

```
trainmod<-traindata
testmod<-testdata

trainmod$Severity=NULL
trainmod$Mild=NULL
trainmod$Sev=NULL
testmod$Severity=NULL
testmod$Mild=NULL
testmod$Sev=NULL

table(trainmod$Mod)
```

```
##
## moderate   others
##      211      552
```

- ***New training data with SMOTE***

```
set.seed(seed)
trainmod.smote <- SMOTE(trainmod[,-1],trainmod$Mod ,K = 5, dup_size=0)
trainmod.smote <- trainmod.smote$data
trainmod.smote$class <- as.factor(trainmod.smote$class)
table(trainmod.smote$class)

##
## moderate   others
##      422      552

registerDoParallel(5)
getDoParWorkers()

## [1] 5
```

- ***Stacking algorithms***

```
ovrctrl <- trainControl(method=”repeatedcv”, number=10, repeats=5,
                        savePredictions=’final’,
                        classProbs=TRUE,
                        summaryFunction = twoClassSummary,
                        allowParallel=TRUE,
                        index = createFolds(trainmod$Mod, 10))

metric <- “ROC”
algorithmList <- c(‘rf’, ‘knn’, ‘svmRadial’, ‘nnet’, ‘xgbTree’)
set.seed(seed)
model_mod <- caretList(Mod~., data=trainmod,
                       metric=metric,
                       trControl=ovrctrl,
                       methodList= algorithmList,
                       tuneList = NULL,
                       continue_on_fail = FALSE)

## # weights:  55
## initial  value 873.602044
## iter  10 value 424.667723
## iter  20 value 383.711368
## iter  30 value 367.517816
## iter  40 value 354.997991
## iter  50 value 350.490980
```

```
## iter  60 value 350.020174
## iter  70 value 349.733714
## iter  80 value 349.620344
## iter  90 value 349.608307
## final  value 349.607786
## converged
```

```
result_mod <- resamples(model_mod)
summary(result_mod)
```

```
##
## Call:
## summary.resamples(object = result_mod)
##
## Models: rf, knn, svmRadial, nnet, xgbTree
## Number of resamples: 10
##
## ROC
##                Min.   1st Qu.    Median      Mean   3rd Qu.      Max. NA's
## rf        0.6635770 0.6831979 0.6961188 0.6948294 0.7038812 0.7224717    0
## knn       0.6185852 0.6443631 0.6573785 0.6563481 0.6718389 0.6904442    0
## svmRadial 0.6250609 0.6591450 0.6686064 0.6704902 0.6813632 0.7096262    0
## nnet      0.5739601 0.6309992 0.6515355 0.6555996 0.6883829 0.7196514    0
## xgbTree   0.5997723 0.6401806 0.6679286 0.6626382 0.6914341 0.7058085    0
##
## Sens
##                 Min.     1st Qu.     Median      Mean   3rd Qu.      Max. N
A's
## rf        0.08947368 0.122368421 0.15526316 0.1490365 0.1690894 0.2000000
   0
## knn       0.11578947 0.168421053 0.19789752 0.2011585 0.2421053 0.3052632
   0
## svmRadial 0.00000000 0.006578947 0.08157895 0.1175327 0.2039474 0.3121693
   0
## nnet      0.23157895 0.305263158 0.35526316 0.3581203 0.4144737 0.4789474
   0
## xgbTree   0.18421053 0.206578947 0.23421053 0.2475132 0.2668755 0.3947368
   0
##
## Spec
##                Min.   1st Qu.    Median      Mean   3rd Qu.      Max. NA's
## rf        0.9455645 0.9527163 0.9617361 0.9615516 0.9708249 0.9758551    0
## knn       0.8450704 0.9024145 0.9133876 0.9126424 0.9285339 0.9517103    0
## svmRadial 0.8588710 0.9300490 0.9668008 0.9552910 0.9979879 1.0000000    0
## nnet      0.7625755 0.7997988 0.8287893 0.8299142 0.8544118 0.9114688    0
## xgbTree   0.8649194 0.8737425 0.9044266 0.9035804 0.9215292 0.9637827    0
```

```
ovrctrl_smote <- trainControl(method="repeatedcv", number=10, repeats=5,
                        savePredictions='final',
```

```
                        classProbs=TRUE,
                        summaryFunction = twoClassSummary,
                        allowParallel=TRUE,
                        index = createFolds(trainmod.smote$class, 10))

set.seed(seed)
model_mod_smote <- caretList(class~., data=trainmod.smote,
                       metric=metric,
                       trControl=ovrctrl_smote,
                       methodList= algorithmList,
                       tuneList = NULL,
                       continue_on_fail = FALSE)

## # weights:  91
## initial  value 702.843816
## iter  10 value 524.799114
## iter  20 value 467.820500
## iter  30 value 442.187527
## iter  40 value 434.942247
## iter  50 value 432.273246
## iter  60 value 429.193347
## iter  70 value 425.651837
## iter  80 value 425.363615
## iter  90 value 425.270337
## iter 100 value 425.235075
## final  value 425.235075
## stopped after 100 iterations

result_mod_smote <- resamples(model_mod_smote)
summary(result_mod_smote)

##
## Call:
## summary.resamples(object = result_mod_smote)
##
## Models: rf, knn, svmRadial, nnet, xgbTree
## Number of resamples: 10
##
## ROC
##                Min.   1st Qu.    Median      Mean   3rd Qu.      Max. NA's
## rf        0.7481018 0.7784311 0.7911944 0.7910262 0.8090266 0.8215689    0
## knn       0.6479112 0.6660410 0.6930857 0.6874171 0.7044228 0.7214824    0
## svmRadial 0.7226204 0.7440408 0.7496411 0.7523656 0.7672764 0.7776792    0
## nnet      0.6828180 0.6946968 0.7208469 0.7204573 0.7347056 0.7659997    0
## xgbTree   0.7371519 0.7498230 0.7588016 0.7626288 0.7650216 0.7964842    0
##
## Sens
##                Min.   1st Qu.    Median      Mean   3rd Qu.      Max. NA's
## rf        0.5000000 0.5565789 0.5994862 0.5924323 0.6250000 0.6868421    0
```

```
## knn       0.5236842 0.5723684 0.5868421 0.6092577 0.6392255 0.7131579    0
## svmRadial 0.3473684 0.4796053 0.5565789 0.5466657 0.6168501 0.7176781    0
## nnet      0.4868421 0.5907895 0.6047424 0.6071712 0.6407895 0.6842105    0
## xgbTree   0.5447368 0.5753923 0.6368421 0.6250701 0.6677701 0.7026316    0
##
## Spec
##                Min.   1st Qu.    Median      Mean   3rd Qu.      Max. NA's
## rf        0.7726358 0.7783374 0.7975819 0.8119868 0.8375252 0.8913481    0
## knn       0.5714286 0.6462038 0.6666795 0.6696846 0.7117706 0.7645875    0
## svmRadial 0.6995968 0.7449698 0.7957746 0.7968882 0.8469993 0.9094567    0
## nnet      0.6512097 0.6921529 0.7132797 0.7175918 0.7494970 0.7802419    0
## xgbTree   0.6915323 0.7243461 0.7474849 0.7489875 0.7728011 0.8148893    0

stctrl <- trainControl(method="repeatedcv", number=10, repeats=5,
                       savePredictions=TRUE,
                       classProbs=TRUE,
                       summaryFunction = twoClassSummary,
                       allowParallel=TRUE,
                       index = createFolds(trainmod$Mod, 10))
```

- ***Combine Predictions with Random Forest***

```
set.seed(seed)
stack.rf_mod <- caretStack(model_mod, method="rf", metric="ROC", trControl=st
ctrl)
print(stack.rf_mod)

## A rf ensemble of 5 base models: rf, knn, svmRadial, nnet, xgbTree
##
## Ensemble results:
## Random Forest
##
## 6867 samples
##    5 predictor
##    2 classes: 'moderate', 'others'
##
## No pre-processing
## Resampling: Cross-Validated (10 fold, repeated 5 times)
## Summary of sample sizes: 76, 76, 76, 78, 76, 76, …
## Resampling results across tuning parameters:
##
##   mtry  ROC        Sens       Spec
##   2     0.6121760  0.2098907  0.8894496
##   3     0.6066516  0.2265888  0.8750516
##   5     0.6013541  0.2358404  0.8634845
##
## ROC was used to select the optimal model using the largest value.
## The final value used for the model was mtry = 2.
```

```
Test_pred_mod_rf <- predict(stack.rf_mod, newdata = testmod)
confusionMatrix(test_pred_mod_rf, testmod$Mod)

## Confusion Matrix and Statistics
##
##             Reference
## Prediction moderate others
##   moderate       18     13
##   others         47    196
##
##                Accuracy : 0.781
##                  95% CI : (0.7273, 0.8285)
##     No Information Rate : 0.7628
##     P-Value [Acc > NIR] : 0.2638
##
##                   Kappa : 0.2619
##
##  Mcnemar’s Test P-Value : 2.042e-05
##
##             Sensitivity : 0.27692
##             Specificity : 0.93780
##          Pos Pred Value : 0.58065
##          Neg Pred Value : 0.80658
##              Prevalence : 0.23723
##          Detection Rate : 0.06569
##    Detection Prevalence : 0.11314
##       Balanced Accuracy : 0.60736
##
##        ‘Positive’ Class : moderate
##
```

- ***Combine Predictions with Generalized Linear Model***

```
set.seed(seed)
stack.glm_mod <- caretStack(model_mod, method=”glm”, metric=”ROC”, trControl=
stctrl)
print(stack.glm_mod)

## A glm ensemble of 5 base models: rf, knn, svmRadial, nnet, xgbTree
##
## Ensemble results:
## Generalized Linear Model
##
## 6867 samples
##    5 predictor
##    2 classes: ‘moderate’, ‘others’
##
## No pre-processing
```

```
## Resampling: Cross-Validated (10 fold, repeated 5 times)
## Summary of sample sizes: 76, 76, 76, 78, 76, 76, …
## Resampling results:
##
##   ROC        Sens       Spec
##   0.6154926  0.1869677  0.9178209
```

```
test_pred_mod_glm <- predict(stack.glm_mod, newdata = testmod)
confusionMatrix(test_pred_mod_glm, testmod$Mod)
```

```
## Confusion Matrix and Statistics
##
##           Reference
## Prediction moderate others
##   moderate       15      8
##   others         50    201
##
##                Accuracy : 0.7883
##                  95% CI : (0.7351, 0.8352)
##     No Information Rate : 0.7628
##     P-Value [Acc > NIR] : 0.1784
##
##                   Kappa : 0.2476
##
##  Mcnemar’s Test P-Value : 7.303e-08
##
##             Sensitivity : 0.23077
##             Specificity : 0.96172
##          Pos Pred Value : 0.65217
##          Neg Pred Value : 0.80080
##              Prevalence : 0.23723
##          Detection Rate : 0.05474
##    Detection Prevalence : 0.08394
##       Balanced Accuracy : 0.59625
##
##        ‘Positive’ Class : moderate
##
```

- ***Combine Predictions with eXtreme Gradient Boosting***

```
set.seed(seed)
stack.xgb_mod <- caretStack(model_mod, method=”xgbTree”, metric=”ROC”, trCont
rol=stctrl)
print(stack.xgb_mod)
```

```
## A xgbTree ensemble of 5 base models: rf, knn, svmRadial, nnet, xgbTree
##
## Ensemble results:
```

```
## eXtreme Gradient Boosting
##
## 6867 samples
##    5 predictor
##    2 classes: 'moderate', 'others'
##
## No pre-processing
## Resampling: Cross-Validated (10 fold, repeated 5 times)
## Summary of sample sizes: 76, 76, 76, 78, 76, 76, …
## Resampling results across tuning parameters:
## Tuned ROC: 0.3  1          0.8                0.50        50      0.6156437
## ## Tuning parameter 'gamma' was held constant at a value of 0
## Tuning
##  parameter 'min_child_weight' was held constant at a value of 1
## ROC was used to select the optimal model using the largest value.
## The final values used for the model were nrounds = 50, max_depth = 1, eta
##  = 0.3, gamma = 0, colsample_bytree = 0.8, min_child_weight = 1 and subsam
ple = 0.5.
```

```
test_pred_mod_xgb <- predict(stack.xgb_mod, newdata = testmod)
confusionMatrix(test_pred_mod_xgb, testmod$Mod)
```

```
## Confusion Matrix and Statistics
##
##             Reference
## Prediction moderate others
##   moderate       11      6
##   others         54    203
##
##                Accuracy : 0.781
##                  95% CI : (0.7273, 0.8285)
##     No Information Rate : 0.7628
##     P-Value [Acc > NIR] : 0.2638
##
##                   Kappa : 0.1885
##
##  Mcnemar's Test P-Value : 1.298e-09
##
##             Sensitivity : 0.16923
##             Specificity : 0.97129
##          Pos Pred Value : 0.64706
##          Neg Pred Value : 0.78988
##              Prevalence : 0.23723
##          Detection Rate : 0.04015
##    Detection Prevalence : 0.06204
##       Balanced Accuracy : 0.57026
##
##        'Positive' Class : moderate
##
```

```
stctrl_smote <- trainControl(method="repeatedcv", number=10, repeats=5,
                        savePredictions=TRUE,
                        classProbs=TRUE,
                        summaryFunction = twoClassSummary,
                        allowParallel=TRUE)
```

- ***Combine Predictions with Random Forest (SMOTE)***

```
set.seed(seed)
stack.rf_mod_smote <- caretStack(model_mod_smote, method="rf", metric="ROC",
trControl=stctrl_smote)
print(stack.rf_mod_smote)

## A rf ensemble of 5 base models: rf, knn, svmRadial, nnet, xgbTree
##
## Ensemble results:
## Random Forest
##
## 8766 samples
##    5 predictor
##    2 classes: 'moderate', 'others'
##
## No pre-processing
## Resampling: Cross-Validated (10 fold, repeated 5 times)
## Summary of sample sizes: 7889, 7889, 7889, 7889, 7889, 7890, …
## Resampling results across tuning parameters:
##
##   mtry  ROC        Sens       Spec
##   2     0.8075300  0.6734652  0.7841787
##   3     0.8056296  0.6733075  0.7817242
##   5     0.8030222  0.6732547  0.7802341
##
## ROC was used to select the optimal model using the largest value.
## The final value used for the model was mtry = 2.

Test_pred_mod_rf_smote <- predict(stack.rf_mod_smote, newdata = testmod)
confusionMatrix(test_pred_mod_rf_smote, testmod$Mod)

## Confusion Matrix and Statistics
##
##           Reference
## Prediction moderate others
##   moderate       39     32
##   others         26    177
##
##                Accuracy : 0.7883
##                  95% CI : (0.7351, 0.8352)
##     No Information Rate : 0.7628
##     P-Value [Acc > NIR] : 0.1784
```

```
##
##                    Kappa : 0.4331
##
##  Mcnemar’s Test P-Value : 0.5115
##
##             Sensitivity : 0.6000
##             Specificity : 0.8469
##          Pos Pred Value : 0.5493
##          Neg Pred Value : 0.8719
##              Prevalence : 0.2372
##          Detection Rate : 0.1423
##    Detection Prevalence : 0.2591
##       Balanced Accuracy : 0.7234
##
##        ‘Positive’ Class : moderate
##
```

- ***Combine Predictions with Generalized Linear Model (SMOTE)***

```
set.seed(seed)
stack.glm_mod_smote <- caretStack(model_mod_smote, method=”glm”, metric=”RO
C”, trControl=stctrl_smote)
print(stack.glm_mod_smote)

## A glm ensemble of 5 base models: rf, knn, svmRadial, nnet, xgbTree
##
## Ensemble results:
## Generalized Linear Model
##
## 8766 samples
##    5 predictor
##    2 classes: ‘moderate’, ‘others’
##
## No pre-processing
## Resampling: Cross-Validated (10 fold, repeated 5 times)
## Summary of sample sizes: 7889, 7889, 7889, 7889, 7889, 7890, …
## Resampling results:
##
##   ROC        Sens       Spec
##   0.7918815  0.6355481  0.7864732

test_pred_mod_glm_smote <- predict(stack.glm_mod_smote, newdata = testmod)
confusionMatrix(test_pred_mod_glm_smote, testmod$Mod)

## Confusion Matrix and Statistics
##
##             Reference
## Prediction moderate others
```

```
##   moderate       35     30
##   others         30    179
##
##                Accuracy : 0.781
##                  95% CI : (0.7273, 0.8285)
##     No Information Rate : 0.7628
##     P-Value [Acc > NIR] : 0.2638
##
##                   Kappa : 0.3949
##
##  Mcnemar’s Test P-Value : 1.0000
##
##             Sensitivity : 0.5385
##             Specificity : 0.8565
##          Pos Pred Value : 0.5385
##          Neg Pred Value : 0.8565
##              Prevalence : 0.2372
##          Detection Rate : 0.1277
##    Detection Prevalence : 0.2372
##       Balanced Accuracy : 0.6975
##
##        ‘Positive’ Class : moderate
##
```

- ***Combine Predictions with eXtreme Gradient Boosting (SMOTE)***

```
set.seed(seed)
stack.xgb_mod_smote <- caretStack(model_mod_smote, method=”xgbTree”, metric=”
ROC”, trControl=stctrl_smote)
print(stack.xgb_mod_smote)
```

```
## A xgbTree ensemble of 5 base models: rf, knn, svmRadial, nnet, xgbTree
##
## Ensemble results:
## eXtreme Gradient Boosting
##
## 8766 samples
##    5 predictor
##    2 classes: ‘moderate’, ‘others’
##
## No pre-processing
## Resampling: Cross-Validated (10 fold, repeated 5 times)
## Summary of sample sizes: 7889, 7889, 7889, 7889, 7889, 7890, …
## Resampling results across tuning parameters:
## Tuned ROC: 0.3  2         0.8               1.00        50      0.7925637
## Tuning parameter ‘gamma’ was held constant at a value of 0
## Tuning
##  parameter ‘min_child_weight’ was held constant at a value of 1
```

```
## ROC was used to select the optimal model using the largest value.
## The final values used for the model were nrounds = 50, max_depth = 2, eta
##  = 0.3, gamma = 0, colsample_bytree = 0.8, min_child_weight = 1 and subsam
ple = 1.
```

```
Test_pred_mod_xgb_smote <- predict(stack.xgb_mod_smote, newdata = testmod)
confusionMatrix(test_pred_mod_xgb_smote, testmod$Mod)
```

```
## Confusion Matrix and Statistics
##
##             Reference
## Prediction moderate others
##   moderate       38     32
##   others         27    177
##
##                Accuracy : 0.7847
##                  95% CI : (0.7312, 0.8319)
##     No Information Rate : 0.7628
##     P-Value [Acc > NIR] : 0.2188
##
##                   Kappa : 0.4204
##
##  Mcnemar's Test P-Value : 0.6025
##
##             Sensitivity : 0.5846
##             Specificity : 0.8469
##          Pos Pred Value : 0.5429
##          Neg Pred Value : 0.8676
##              Prevalence : 0.2372
##          Detection Rate : 0.1387
##    Detection Prevalence : 0.2555
##       Balanced Accuracy : 0.7158
##
##        'Positive' Class : moderate
##
```

■ *Data extraction for severe-versus-rest classification*

```
trainsev<-traindata
testsev<-testdata

trainsev$Severity=NULL
trainsev$Mild=NULL
trainsev$Mod=NULL
testsev$Severity=NULL
```

```
testsev$Mild=NULL
testsev$Mod=NULL

trainsev$Sev<-ifelse(trainsev$Sev=="severe", 'X1','X2')
prop.table(table(trainsev$Sev))

##
##        X1        X2
## 0.2385321 0.7614679

testsev$Sev<-ifelse(testsev$Sev=="severe", 'X1','X2')
prop.table(table(testsev$Sev))

##
##        X1        X2
## 0.2627737 0.7372263

trainsev$Sev<-as.factor(trainsev$Sev)
testsev$Sev<-as.factor(testsev$Sev)

table(trainsev$Sev)

##
##  X1  X2
## 182 581

table(testsev$Sev)

##
##  X1  X2
##  72 202
```

- ***New training data with SMOTE***

```
set.seed(seed)
trainsev.smote <- SMOTE(trainsev[,-1],trainsev$Sev ,K = 5, dup_size=0)
trainsev.smote <- trainsev.smote$data
trainsev.smote$class <- as.factor(trainsev.smote$class)
table(trainsev.smote$class)

##
##  X1  X2
## 546 581

registerDoParallel(5)
getDoParWorkers()

## [1] 5
```

- ***Stacking algorithms***

```
ovrctrl <- trainControl(method="repeatedcv", number=10, repeats=5,
                        savePredictions='final',
                        classProbs=TRUE,
                        summaryFunction = twoClassSummary,
                        allowParallel=TRUE,
                        index = createFolds(trainsev$Sev, 10))
metric <- "ROC"
algorithmList <- c('rf', 'knn', 'svmRadial', 'nnet', 'xgbTree')
set.seed(seed)
model_sev <- caretList(Sev~., data=trainsev,
                       metric=metric,
                       trControl=ovrctrl,
                       methodList= algorithmList,
                       tuneList = NULL,
                       continue_on_fail = FALSE)

## # weights:  19
## initial  value 431.748172
## iter  10 value 233.415971
## iter  20 value 199.409416
## iter  30 value 195.619305
## iter  40 value 195.522573
## final  value 195.522454
## converged

result_sev <- resamples(model_sev)
summary(result_sev)

##
## Call:
## summary.resamples(object = result_sev)
##
## Models: rf, knn, svmRadial, nnet, xgbTree
## Number of resamples: 10
##
## ROC
##                Min.   1st Qu.    Median      Mean   3rd Qu.      Max. NA's
## rf        0.9089388 0.9181515 0.9189002 0.9210513 0.9251300 0.9365177    0
## knn       0.8586959 0.8852277 0.8907831 0.8891149 0.8940206 0.9136606    0
## svmRadial 0.8802115 0.8977566 0.9014331 0.9033204 0.9126143 0.9212389    0
## nnet      0.8674218 0.8847643 0.9032875 0.8981911 0.9094660 0.9238388    0
## xgbTree   0.8777806 0.9075979 0.9109577 0.9098654 0.9174060 0.9240428    0
##
## Sens
##                Min.   1st Qu.    Median      Mean   3rd Qu.      Max. NA's
## rf        0.5914634 0.6158537 0.6360355 0.6398287 0.6692073 0.6993865    0
## knn       0.3742331 0.4695122 0.5091463 0.5103659 0.5716463 0.6257669    0
## svmRadial 0.6524390 0.6763617 0.7073171 0.7100105 0.7389084 0.7865854    0
```

```
## nnet      0.6341463 0.6875935 0.6951220 0.7063594 0.7366265 0.7621951    0
## xgbTree   0.5670732 0.6366527 0.6554878 0.6587573 0.6905488 0.7177914    0
##
## Spec
##                Min.   1st Qu.    Median      Mean   3rd Qu.      Max. NA's
## rf        0.9560229 0.9588910 0.9722753 0.9695941 0.9770445 0.9847036    0
## knn       0.9349904 0.9679732 0.9760994 0.9713171 0.9804015 0.9885057    0
## svmRadial 0.9196941 0.9364245 0.9483253 0.9485561 0.9627151 0.9770554    0
## nnet      0.8852772 0.8972275 0.9196208 0.9183410 0.9345124 0.9598470    0
## xgbTree   0.9120459 0.9273423 0.9416826 0.9433943 0.9593461 0.9770554    0
```

```
ovrctrl_smote <- trainControl(method="repeatedcv", number=10, repeats=5,
                        savePredictions='final',
                        classProbs=TRUE,
                        summaryFunction = twoClassSummary,
                        allowParallel=TRUE,
                        index = createFolds(trainsev.smote$class, 10))

set.seed(seed)
model_sev_smote <- caretList(class~., data=trainsev.smote,
                       metric=metric,
                       trControl=ovrctrl_smote,
                       methodList= algorithmList,
                       tuneList = NULL,
                       continue_on_fail = FALSE)
```

```
## # weights:  91
## initial  value 792.366080
## iter  10 value 321.947857
## iter  20 value 283.340624
## iter  30 value 264.500386
## iter  40 value 253.933547
## iter  50 value 248.809847
## iter  60 value 246.281311
## iter  70 value 245.432540
## iter  80 value 244.916189
## iter  90 value 244.594949
## iter 100 value 244.555753
## final  value 244.555753
## stopped after 100 iterations
```

```
result_sev_smote <- resamples(model_sev_smote)
summary(result_sev_smote)
```

```
##
## Call:
## summary.resamples(object = result_sev_smote)
##
## Models: rf, knn, svmRadial, nnet, xgbTree
## Number of resamples: 10
```

```
##
## ROC
##                 Min.   1st Qu.    Median      Mean   3rd Qu.      Max. NA's
## rf        0.9395408 0.9414188 0.9466838 0.9468358 0.9514308 0.9555041    0
## knn       0.9101373 0.9140514 0.9205313 0.9191597 0.9239102 0.9265980    0
## svmRadial 0.9115319 0.9198564 0.9247045 0.9232215 0.9264164 0.9326548    0
## nnet      0.8762388 0.9198770 0.9248591 0.9191346 0.9300964 0.9342287    0
## xgbTree   0.9232611 0.9306304 0.9363792 0.9342979 0.9384096 0.9437870    0
##
## Sens
##                 Min.   1st Qu.    Median      Mean   3rd Qu.      Max. NA's
## rf        0.7841141 0.8018293 0.8280761 0.8237639 0.8309572 0.8800813    0
## knn       0.7922607 0.8335869 0.8433428 0.8390252 0.8539462 0.8638211    0
## svmRadial 0.7556008 0.7803822 0.8026406 0.8091020 0.8345214 0.8841463    0
## nnet      0.7800407 0.8310369 0.8574338 0.8571175 0.8795080 0.9268293    0
## xgbTree   0.7983707 0.8322332 0.8382511 0.8445209 0.8482688 0.9044715    0
##
## Spec
##                 Min.   1st Qu.    Median      Mean   3rd Qu.      Max. NA's
## rf        0.8833652 0.9053153 0.9292543 0.9235028 0.9397706 0.9560229    0
## knn       0.8298279 0.8360421 0.8516663 0.8525512 0.8675908 0.8833652    0
## svmRadial 0.8107075 0.8508604 0.8880373 0.8831524 0.9158700 0.9445507    0
## nnet      0.7801147 0.8150096 0.8298279 0.8328469 0.8408222 0.8929254    0
## xgbTree   0.8451243 0.8583026 0.8795411 0.8802785 0.8900574 0.9388145    0
```

```
stctrl <- trainControl(method="repeatedcv", number=10, repeats=5,
                       savePredictions=TRUE,
                       classProbs=TRUE,
                       summaryFunction = twoClassSummary,
                       allowParallel=TRUE,
                       index = createFolds(trainsev$Sev, 10))
```

- ***Combine Predictions with Random Forest***

```
set.seed(seed)
stack.rf_sev <- caretStack(model_sev, method="rf", metric="ROC", trControl=st
ctrl)
print(stack.rf_sev)
```

```
## A rf ensemble of 5 base models: rf, knn, svmRadial, nnet, xgbTree
##
## Ensemble results:
## Random Forest
##
## 6867 samples
##    5 predictor
##    2 classes: 'X1', 'X2'
##
## No pre-processing
```

```
## Resampling: Cross-Validated (10 fold, repeated 5 times)
## Summary of sample sizes: 76, 76, 76, 77, 76, 76, ...
## Resampling results across tuning parameters:
##
##   mtry  ROC        Sens       Spec
##   2     0.9081084  0.8167290  0.8632374
##   3     0.9068018  0.8153099  0.8597151
##   5     0.9029896  0.7881151  0.8576454
##
## ROC was used to select the optimal model using the largest value.
## The final value used for the model was mtry = 2.
```

```
test_pred_sev_rf <- predict(stack.rf_sev, newdata = testmod)
confusionMatrix(test_pred_sev_rf, testsev$Sev)
```

```
## Confusion Matrix and Statistics
##
##           Reference
## Prediction  X1  X2
##         X1  50   3
##         X2  22 199
##
##                Accuracy : 0.9088
##                  95% CI : (0.8683, 0.9401)
##     No Information Rate : 0.7372
##     P-Value [Acc > NIR] : 8.417e-13
##
##                   Kappa : 0.7427
##
##  Mcnemar's Test P-Value : 0.0003182
##
##             Sensitivity : 0.6944
##             Specificity : 0.9851
##          Pos Pred Value : 0.9434
##          Neg Pred Value : 0.9005
##              Prevalence : 0.2628
##          Detection Rate : 0.1825
##    Detection Prevalence : 0.1934
##       Balanced Accuracy : 0.8398
##
##        'Positive' Class : X1
##
```

- ***Combine Predictions with Generalized Linear Model***

```
set.seed(seed)
stack.glm_sev <- caretStack(model_sev, method="glm", metric="ROC", trControl=
```

```
stctrl)
print(stack.glm_sev)
```

```
## A glm ensemble of 5 base models: rf, knn, svmRadial, nnet, xgbTree
##
## Ensemble results:
## Generalized Linear Model
##
## 6867 samples
##    5 predictor
##    2 classes: 'X1', 'X2'
##
## No pre-processing
## Resampling: Cross-Validated (10 fold, repeated 5 times)
## Summary of sample sizes: 76, 76, 76, 77, 76, 76, ...
## Resampling results:
##
##   ROC        Sens       Spec
##   0.8936162  0.7952405  0.8400553
```

```
test_pred_sev_glm <- predict(stack.glm_sev, newdata = testmod)
confusionMatrix(test_pred_sev_glm, testsev$Sev)
```

```
## Confusion Matrix and Statistics
##
##           Reference
## Prediction  X1  X2
##         X1  50   4
##         X2  22 198
##
##                Accuracy : 0.9051
##                  95% CI : (0.8641, 0.9371)
##     No Information Rate : 0.7372
##     P-Value [Acc > NIR] : 2.922e-12
##
##                   Kappa : 0.7337
##
##  Mcnemar's Test P-Value : 0.0008561
##
##             Sensitivity : 0.6944
##             Specificity : 0.9802
##          Pos Pred Value : 0.9259
##          Neg Pred Value : 0.9000
##              Prevalence : 0.2628
##          Detection Rate : 0.1825
##    Detection Prevalence : 0.1971
##       Balanced Accuracy : 0.8373
##
```

```
##        'Positive' Class : X1
##
```

- ***Combine Predictions with eXtreme Gradient Boosting***

```
set.seed(seed)
stack.xgb_sev <- caretStack(model_sev, method="xgbTree", metric="ROC", trCont
rol=stctrl)
print(stack.xgb_sev)

## A xgbTree ensemble of 5 base models: rf, knn, svmRadial, nnet, xgbTree
##
## Ensemble results:
## eXtreme Gradient Boosting
##
## 6867 samples
##    5 predictor
##    2 classes: 'X1', 'X2'
##
## No pre-processing
## Resampling: Cross-Validated (10 fold, repeated 5 times)
## Summary of sample sizes: 76, 76, 76, 77, 76, 76, ...
## Resampling results across tuning parameters:
## Tuned ROC: 0.3  1          0.6              0.75        50      0.8964524

## Tuning parameter 'gamma' was held constant at a value of 0
## Tuning parameter 'min_child_weight' was held constant at a value of 1
## ROC was used to select the optimal model using the largest value.
## The final values used for the model were nrounds = 50, max_depth = 1, eta
##  = 0.3, gamma = 0, colsample_bytree = 0.6, min_child_weight = 1 and subsam
ple = 0.75.

test_pred_sev_xgb <- predict(stack.xgb_sev, newdata = testmod)
confusionMatrix(test_pred_sev_xgb, testsev$Sev)

## Confusion Matrix and Statistics
##
##           Reference
## Prediction  X1  X2
##         X1  48   4
##         X2  24 198
##
##                Accuracy : 0.8978
##                  95% CI : (0.8557, 0.931)
##     No Information Rate : 0.7372
##     P-Value [Acc > NIR] : 3.115e-11
##
##                   Kappa : 0.7104
```

```
##
##  Mcnemar's Test P-Value : 0.0003298
##
##             Sensitivity : 0.6667
##             Specificity : 0.9802
##          Pos Pred Value : 0.9231
##          Neg Pred Value : 0.8919
##              Prevalence : 0.2628
##          Detection Rate : 0.1752
##    Detection Prevalence : 0.1898
##       Balanced Accuracy : 0.8234
##
##        'Positive' Class : X1
##
```

```
stctrl_smote <- trainControl(method="repeatedcv", number=10, repeats=5,
                       savePredictions=TRUE,
                       classProbs=TRUE,
                       summaryFunction = twoClassSummary,
                       allowParallel=TRUE)
```

- ***Combine Predictions with Random Forest (SMOTE)***

```
set.seed(seed)
stack.rf_sev_smote <- caretStack(model_sev_smote, method="rf", metric="ROC",
trControl=stctrl_smote)
print(stack.rf_sev_smote)
```

```
## A rf ensemble of 5 base models: rf, knn, svmRadial, nnet, xgbTree
##
## Ensemble results:
## Random Forest
##
## 10143 samples
##     5 predictor
##     2 classes: 'X1', 'X2'
##
## No pre-processing
## Resampling: Cross-Validated (10 fold, repeated 5 times)
## Summary of sample sizes: 9129, 9128, 9128, 9128, 9128, 9130, ...
## Resampling results across tuning parameters:
##
##   mtry  ROC        Sens       Spec
##   2     0.9562142  0.8751297  0.9058715
##   3     0.9552654  0.8759848  0.9042647
##   5     0.9539610  0.8754964  0.9025439
##
## ROC was used to select the optimal model using the largest value.
## The final value used for the model was mtry = 2.
```

```r
test_pred_sev_rf_smote <- predict(stack.rf_sev_smote, newdata = testsev)
confusionMatrix(test_pred_sev_rf_smote, testsev$Sev)
```

```
## Confusion Matrix and Statistics
##
##           Reference
## Prediction  X1  X2
##         X1  48   5
##         X2  24 197
##
##                Accuracy : 0.8942
##                  95% CI : (0.8515, 0.928)
##     No Information Rate : 0.7372
##     P-Value [Acc > NIR] : 9.59e-11
##
##                   Kappa : 0.7015
##
##  Mcnemar's Test P-Value : 0.0008302
##
##             Sensitivity : 0.6667
##             Specificity : 0.9752
##          Pos Pred Value : 0.9057
##          Neg Pred Value : 0.8914
##              Prevalence : 0.2628
##          Detection Rate : 0.1752
##    Detection Prevalence : 0.1934
##       Balanced Accuracy : 0.8210
##
##        'Positive' Class : X1
##
```

- ***Combine Predictions with Generalized Linear Model (SMOTE)***

```r
set.seed(seed)
stack.glm_sev_smote <- caretStack(model_sev_smote, method="glm", metric="ROC
", trControl=stctrl_smote)
print(stack.glm_sev_smote)
```

```
## A glm ensemble of 5 base models: rf, knn, svmRadial, nnet, xgbTree
##
## Ensemble results:
## Generalized Linear Model
##
## 10143 samples
##     5 predictor
##     2 classes: 'X1', 'X2'
##
## No pre-processing
```

```
## Resampling: Cross-Validated (10 fold, repeated 5 times)
## Summary of sample sizes: 9129, 9128, 9128, 9128, 9128, 9130, ...
## Resampling results:
##
##   ROC       Sens       Spec
##   0.945783  0.8490824  0.8998275
```

```
test_pred_sev_glm_smote <- predict(stack.glm_sev_smote, newdata = testsev)
confusionMatrix(test_pred_sev_glm_smote, testsev$Sev)
```

```
## Confusion Matrix and Statistics
##
##           Reference
## Prediction  X1  X2
##         X1  50   5
##         X2  22 197
##
##                Accuracy : 0.9015
##                  95% CI : (0.8599, 0.9341)
##     No Information Rate : 0.7372
##     P-Value [Acc > NIR] : 9.733e-12
##
##                   Kappa : 0.7248
##
##  Mcnemar's Test P-Value : 0.002076
##
##             Sensitivity : 0.6944
##             Specificity : 0.9752
##          Pos Pred Value : 0.9091
##          Neg Pred Value : 0.8995
##              Prevalence : 0.2628
##          Detection Rate : 0.1825
##    Detection Prevalence : 0.2007
##       Balanced Accuracy : 0.8348
##
##        'Positive' Class : X1
##
```

- ***Combine Predictions with eXtreme Gradient Boosting (SMOTE)***

```
set.seed(seed)
stack.xgb_sev_smote <- caretStack(model_sev_smote, method="xgbTree", metric="
ROC", trControl=stctrl_smote)
print(stack.xgb_sev_smote)
```

```
## A xgbTree ensemble of 5 base models: rf, knn, svmRadial, nnet, xgbTree
##
## Ensemble results:
```

```
## eXtreme Gradient Boosting
##
## 10143 samples
##     5 predictor
##     2 classes: 'X1', 'X2'
##
## No pre-processing
## Resampling: Cross-Validated (10 fold, repeated 5 times)
## Summary of sample sizes: 9129, 9128, 9128, 9128, 9128, 9130, ...
## Resampling results across tuning parameters:
## Tuned ROC: 0.3  3          0.6                1.00       150      0.9498693
## Tuning parameter 'gamma' was held constant at a value of 0
## Tuning
##  parameter 'min_child_weight' was held constant at a value of 1
## ROC was used to select the optimal model using the largest value.
## The final values used for the model were nrounds = 150, max_depth = 3, eta
##  = 0.3, gamma = 0, colsample_bytree = 0.6, min_child_weight = 1 and subsam
ple = 1.
```

```
test_pred_sev_xgb_smote <- predict(stack.xgb_sev_smote, newdata = testsev)
confusionMatrix(test_pred_sev_xgb_smote, testsev$Sev)
```

```
## Confusion Matrix and Statistics
##
##           Reference
## Prediction  X1  X2
##         X1  53   6
##         X2  19 196
##
##                Accuracy : 0.9088
##                  95% CI : (0.8683, 0.9401)
##     No Information Rate : 0.7372
##     P-Value [Acc > NIR] : 8.417e-13
##
##                   Kappa : 0.75
##
##  Mcnemar's Test P-Value : 0.0164
##
##             Sensitivity : 0.7361
##             Specificity : 0.9703
##          Pos Pred Value : 0.8983
##          Neg Pred Value : 0.9116
##              Prevalence : 0.2628
##          Detection Rate : 0.1934
##    Detection Prevalence : 0.2153
##       Balanced Accuracy : 0.8532
##
##        'Positive' Class : X1
##
```
